# Effects of elevated systolic blood pressure on ischemic heart disease: a Burden of Proof study

**DOI:** 10.1038/s41591-022-01974-1

**Published:** 2022-10-10

**Authors:** Christian Razo, Catherine A. Welgan, Catherine O. Johnson, Susan A. McLaughlin, Vincent Iannucci, Anthony Rodgers, Nelson Wang, Kate E. LeGrand, Reed J. D. Sorensen, Jiawei He, Peng Zheng, Aleksandr Y. Aravkin, Simon I. Hay, Christopher J. L. Murray, Gregory A. Roth

**Affiliations:** 1 Institute for Health Metrics and Evaluation, University of Washington, Seattle, WA USA; 2 Department of Health Metrics Sciences, School of Medicine, University of Washington, Seattle, WA USA; 3 Johns Hopkins University School of Medicine, Baltimore, MD USA; 4 The George Institute for Global Health, The University of New South Wales, Sydney, New South Wales Australia; 5 Department of Applied Mathematics, University of Washington, Seattle, WA USA; 6 Division of Cardiology, University of Washington, Seattle, WA USA

**Keywords:** Risk factors, Cardiovascular diseases

## Abstract

High systolic blood pressure (SBP) is a major risk factor for ischemic heart disease (IHD), the leading cause of death worldwide. Using data from published observational studies and controlled trials, we estimated the mean SBP–IHD dose–response function and burden of proof risk function (BPRF), and we calculated a risk outcome score (ROS) and corresponding star rating (one to five). We found a very strong, significant harmful effect of SBP on IHD, with a mean risk—relative to that at 100 mm Hg SBP—of 1.39 (95% uncertainty interval including between-study heterogeneity 1.34–1.44) at 120 mm Hg, 1.81 (1.70–1.93) at 130 mm Hg and 4.48 (3.81–5.26) at 165 mm Hg. The conservative BPRF measure indicated that SBP exposure between 107.5 and 165.0 mm Hg raised risk by 101.36% on average, yielding a ROS of 0.70 and star rating of five. Our analysis shows that IHD risk was already increasing at 120 mm Hg SBP, rising steadily up to 165 mm Hg and increasing less steeply above that point. Our study endorses the need to prioritize and strengthen strategies for screening, to raise awareness of the need for timely diagnosis and treatment of hypertension and to increase the resources allocated for understanding primordial prevention of elevated blood pressure.

## Abstract (web summary)

A systematic review using the burden of proof meta-analytic method found a significant harmful effect between high systolic blood pressure and ischemic heart disease.

## Main

High SBP is a common modifiable risk factor for IHD^1,2^. IHD is the leading cause of death and disability worldwide, accounting for an estimated 9.1 million (95% uncertainty interval (UI) = 8.4–9.7 million) deaths, 197 million (178–219) prevalent cases and 182 million (170–194) disability-adjusted life years in 2019 (ref. ^3^).

The association between SBP and IHD is one of the most widely investigated health risk–outcome relationships, with substantial evidence for causation^4^. Prospective cohort studies have reported a continuous log-linear association between usual SBP and mortality due to vascular events across diverse population groups with and without pre-existing cardiovascular disease^5–7^. Similarly, double-blinded randomized control trials (RCTs) that examine blood pressure-lowering drugs^8,9^ and meta-analyses of RCT data have provided evidence of the protective effects of pharmacologically induced blood pressure reduction^10,11^.

Despite the extensive body of evidence indicating that elevated SBP is related to increased risk of IHD, several questions and methodological challenges remain unaddressed^12,13^. Trials performed thus far have primarily focused on individuals diagnosed with hypertension or those who are already at high cardiovascular risk, typically involving participants who are known to be at higher levels of risk. Moreover, there are crucial questions about the level at which SBP values should be considered elevated, and whether pharmacological reduction of BP reduces IHD risk even for individuals with relatively low baseline levels^14–16^. Conversely there has been discussion of whether reduction of BP below a certain point may actually increase IHD risk, based on the J-shaped SBP–IHD relationship observed in analyses performed in a subset of cohort studies^17–19^.

Investigation of SBP as a continuous risk function is helpful in the face of multiple current clinical guidelines for BP that suggest different thresholds for applying diagnoses. The American College of Cardiology/American Heart Association 2017 guidelines define normal BP as <120/80 mm Hg, with elevated BP considered to be SBP of 120–129 mm Hg and hypertension defined as SBP ≥130/80 mm Hg (ref. ^20^). In contrast, the European Society of Cardiology/European Society of Hypertension 2018 guidelines consider normal BP to be <130/85 mm Hg, with high-normal defined as BP of 130–139/85–89 mm Hg and hypertension defined as ≥140 mm Hg (ref. ^21^). The International Society of Hypertension 2020 guidelines similarly consider hypertension to be BP ≥140/90 mm Hg (ref. ^22^).

The increasing disease burden attributable to high BP levels worldwide^2^ and the inconsistent global progress in hypertension treatment, control and prevention^23^ demonstrate the relevance of studies that quantify the strength of the evidence using an objective, quantitative, comprehensive and comparative framework^24^. Findings from such studies can be used to support (1) strengthening of targeted and population-based screening strategies that promote hypertension awareness and timely diagnosis; (2) scaling up of effective hypertension treatment to achieve universal coverage; (3) optimization of patient care and follow-up; and (4) increasing resources allocated for primordial prevention and effective treatment of hypertension and IHD from early childhood through the life course.

In this study we assessed the continuous dose–response relationship of SBP to IHD, applying a Bayesian meta-analytic approach^24,25^. We estimated the shape of the risk function and BPRF—defined as a conservative assessment of the effect of SBP on IHD—based on the evidence from both observational cohort studies and RCTs. We included potential covariates related to study design to account for potential bias. Differences in ranges of SBP levels across studies were handled by integration across the risk curve. We also tested for the presence of publication and reporting bias in the data analyzed^24,26,27^. In addition, we minimized the number of a priori statistical assumptions and approximations frequently used in previous meta-analyses.

From the BPRF we computed a ROS, with a higher positive value corresponding to a higher average effect size across SBP values in the data-dense area between the 15th and 85th percentile of SBP distribution in the data analyzed, and thus stronger evidence for the estimated relationship. For further interpretability we converted the ROS into a star rating, with one star indicating no relationship between risk and outcome and five stars indicating a large effect and strong evidence. The ROS offers a method for comparison of the strength of evidence for the effect of SBP on IHD with evidence for other risk factors and outcomes^24^, and a robust approach to effective translation of scientific knowledge into strategic policies for prevention and control of hypertension.

Our results represent an updated systematic synthesis and meta-analysis of the available causal evidence from RCTs and cohort studies examining the effect of SBP on IHD, and show a significant and direct dose–response relationship between SBP levels and IHD risk across all SBP exposure values (100–200 mm Hg), without evidence for a J-shape. Additionally, our findings suggest that IHD risk remains highest for individuals living with the highest SBP levels, providing evidence in support of public health and clinical interventions that will allow people to maintain SBP levels associated with low IHD risk throughout their lives. Table 1 summarizes the main findings, limitations and policy implications of this study.

**Table 1:** Policy summary

Background	While the association between SBP and IHD is one of the most widely investigated risk–outcome relationships, with substantial evidence for causation, there remain questions concerning the level at which SBP should be considered elevated and the consequences of reducing SBP in individuals with relatively low baseline levels—that is, whether the SBP–IHD relationship is J-shaped. In addition, few attempts have been made to unify the available evidence from cohort studies involving the natural history of gradually increasing blood pressure and RCTs that entail lowering of blood pressure through pharmacotherapy.
Main findings and limitations	Our results show a significant and direct dose–response relationship between SBP levels and IHD risk, without evidence for a J-shaped curve. Based on conventional mean RR measures, SBP values of 120 and 130 mm Hg were associated with 39 and 81% higher risk of IHD, respectively, relative to that at 100 mm Hg, while an SBP of 165 mm Hg was associated with 348% higher risk. Similarly, the highly conservative BPRF measure introduced by Zheng et al.^24^ still yielded a robust dose–response relationship, showing a 101.36% increase in IHD risk on average across the data-dense area of the SBP exposure range between 107.5 and 165.0 mm Hg (relative to 100 mm Hg). In the BPRF framework this yielded a five-star rating, signifying strong and consistent evidence supporting the statistically significant association between SBP and IHD. Primary limitations of the present study include (1) the low number of published studies involving participants with either lower or extremely high SBP values; (2) variability among outcome definitions, including RR estimates for different combinations of the fatal and nonfatal endpoints of IHD; and (3) a cutoff date of April 2020 for our literature search.
Policy implications	Even using the highly conservative BPRF approach to evaluate the effect size and strength of the evidence, our findings robustly confirm that high SBP substantially increases the risk of IHD. Our results additionally (1) show that increased IHD risk should be expected at levels defined by some current guidelines as normal or high-normal; (2) suggest it is unlikely that reducing SBP at the low end of the exposure range can increase IHD risk; and (3) provide useful insights on the assumption of risk reversibility. Using the BPRF framework provides a simple way to translate evidence into both clinical practice and policy, and to communicate health risks to the general population. Overall, our results are a call to the health community to (1) prioritize the prevention and control of hypertension, highlighting the need to enhance existing community screening programs to raise hypertension awareness and support timely diagnosis; (2) scale up effective treatment of hypertension to achieve universal coverage of hypertension treatment; and (3) increase the technical capacity and resources allocated for primordial, primary and secondary hypertension and IHD prevention from early childhood through the life course.

## Results

### Systematic review

**Extended Data Fig. 1:**
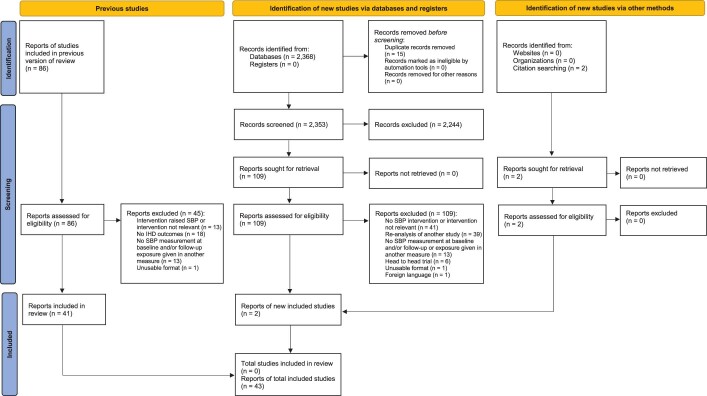
PRISMA flow diagram of systolic blood pressure and ischemic heart disease data seeking approach. The PRISMA flow diagram covering systolic blood pressure and ischemic heart disease. Template is from: Page MJ, McKenzie JE, Bossuyt PM, Boutron I, Hoffmann TC, Mulrow CD, et al. The PRISMA 2020 statement: an updated guideline for reporting systematic reviews. BMJ 2021;372:n71. doi: 10.1136/bmj.n71. For more information, visit: http://www.prisma-statement.org/.

**Extended Data Fig. 2:**
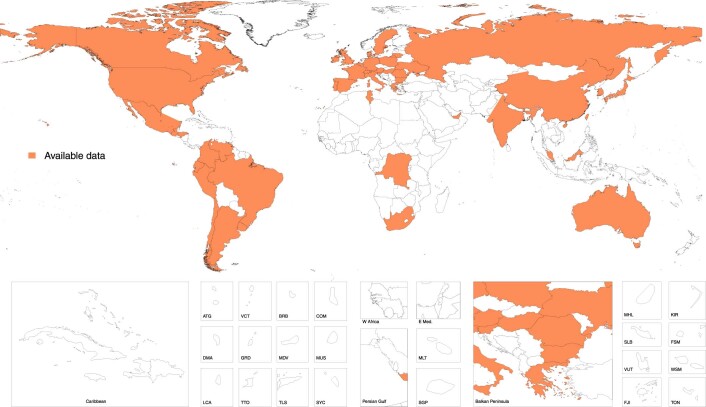
Map of countries where input studies were conducted.

Following Preferred Reporting Items for Systematic Reviews and Meta-Analyses (PRISMA) guidelines^28^ (Supplementary Tables 1 and 2), we identified 86 RCTs from a previous meta-analysis^29^ and 2,368 RCTs published between February 2018 and April 2020. In addition, two studies previously used in the Global Burden of Disease study 2019 (ref. ^2^) to estimate the burden attributable to high SBP were identified. In total, we extracted data from 41 studies: one observational study^30^, one pooled cohort that included 61 prospective studies^31^ and 39 RCTs investigating the effect of blood pressure-lowering drugs on SBP^32–70^ (Extended Data Fig. 1). The data included represent 1,486,007 unique participants and 12,628 IHD events. Studies were conducted across 66 countries (Extended Data Fig. 2). Table 2 summarizes additional characteristics of the studies included. The full citation list of studies included is available in Supplementary Table 3.

**Table 2:** Study characteristics

Study name	Author and year of publication	Population	Average follow-up (years)	Age (years)	Endpoints	Outcome definition	Intervention group	Control group
ABCD-N	Schrier et al., 2002 (ref. ^62^)	Normotensive type 2 diabetic subjects identified from healthcare systems	5.3	40–74	Incidence and mortality	Myocardial infarction and heart failure	Nisoldipine or enalapril	Placebo
ACCORD, Action to Control Cardiovascular Risk in Diabetes Study	ACCORD Study Group, 2010 (ref. ^32^)	High-risk participants with type 2 diabetes	4.7	40–79	Incidence and mortality	Myocardial infarction and coronary heart disease	Intensive therapy	Standard therapy
ACTION Trial	Poole-Wilson, 2004 (ref. ^60^)	Ambulatory patients diagnosed with angina pectoris with and without history of myocardial infarction	6	35–99	Incidence and mortality	Myocardial infarction, angina and heart failure	Nifedipine	Placebo
Active I	Active I Investigators, 2011 (ref. ^33^)	Patients with atrial fibrillation and history of cardiovascular disease (CVD) or hypertension before the study	4.1	75+	Incidence and mortality	Myocardial infarction and heart failure	Irbesartan 150 and 300 mg d^–1^	Placebo
ADVANCE	Patel et al., 2007 (ref. ^58^)	Patients diagnosed with type 2 diabetes mellitus at the age of 30 years or older and with history of major cardiovascular disease or at least one other risk factor for cardiovascular disease	5	55–76	Incidence and mortality	Coronary heart disease	Perindopril 2 mg and indapamide 625 mg	Placebo
CAMELOT	Nissen et al., 2004 (ref. ^56^)	Individuals requiring coronary angiography for evaluation of chest pain or percutaneous coronary intervention with normal blood pressure, and without treatment and without heart failure	2	30–79	Incidence and mortality	Myocardial infarction and angina	Amlodipine or enalapril	Placebo
CARDIO-SIS	Verdecchia et al., 2009 (ref. ^69^)	Patients with systolic blood pressure 150 mm Hg or higher, receiving antihypertensive treatment for at least 12 weeks and without diabetes	2	55+	Incidence and mortality	Myocardial infarction and heart failure	Tight control (<130 mm Hg) of SBP	Usual control (<140 mm Hg) of SBP
DIABHYCAR	Marre et al., 2004 (ref. ^52^)	Individuals with type 2 diabetes and urinary albumin excretion ≥20 mg l^–1^	4	52–78	Incidence and mortality	Myocardial infarction and heart failure	Ramipril	Placebo
DREAM, Diabetes REduction Assessment with ramipril and rosiglitazone Medication	DREAM Trial Investigators, 2006 (ref. ^40^)	People with impaired fasting plasma glucose or impaired glucose tolerance and without diabetes or cardiovascular disease	3	30+	Incidence and mortality	Myocardial infarction, heart failure and angina	Ramipril	Placebo
Dutch TIA	The Dutch TIA Trial Study Group, 1993 (ref. ^67^)	Patients who were seen by a neurologist in one of the 56 collaborating centers and who had a transient ischemic attack or nondisabling ischemic stroke	2.7	18+	Incidence and mortality	Coronary heart disease	Atenolol	Placebo
EUROPA, EUropean trial on Reduction of cardiac events with Perindopril in patients with stable coronary artery disease study	Fox et al., 2003 (ref. ^41^)	Patients with evidence of coronary heart disease and without heart failure	4.2	45–75	Incidence	Myocardial infarction	Perindopril	Placebo
EWPHE, European Working Party on High blood pressure in the Elderly	Amery et al., 1985 (ref. ^34^)	Patients with systolic blood pressure within the limits 160–239 mm Hg and without CVD	4.6	60+	Mortality	Coronary heart disease	Hydrochlorothiazide + triamterene	Placebo
FEVER Felodipine Event Reduction Study	Liu et al., 2005 (ref. ^48^)	Individuals with systolic blood pressure 210 mm Hg or less and DBP <115 mm Hg if under antihypertensive treatment; or systolic blood pressure 160–210 mm Hg or DBP 95–115 mm Hg if untreated	3.3	50–79	Incidence and mortality	Coronary heart disease	Felodipine	Placebo
HOPE-3, Heart Outcomes Prevention Evaluation study 3	Lonn et al., 2016 (ref. ^49^)	Individuals without cardiovascular disease and with at least one of the following cardiovascular risk factors: elevated waist-to-hip ratio, history of low concentration of high-density lipoprotein cholesterol, current or recent tobacco use, dysglycemia, family history of premature coronary disease and mild renal dysfunction	5.6	55+	Incidence and mortality	Myocardial infarction, heart failure and angina and revascularization	Candesartan + hydrochlorothiazide	Placebo
HOPE, Heart Outcomes Prevention Evaluation study	Heart Outcomes Prevention Evaluation Study Investigators, 2000 (ref. ^44^)	Individuals with history of cardiovascular disease and/or diabetes plus at least one other cardiovascular risk factor (hypertension, elevated cholesterol levels, cigarette smoking or microalbuminuria)	5.6	55+	Incidence and mortality	Myocardial infarction	Ramipril 2.5 mg	Placebo
HOT, Hypertension Optimal Treatment	Hannson et al., 1998 (ref. ^43^)	Patients with hypertension and DBP 100–115 mm Hg	3.8	50–80	Incidence and mortality	Myocardial infarction	Diastolic control target <80 mm Hg	Placebo, diastolic control target <90 mm Hg
HYVET	Beckett et al., 2008 (ref. ^36^)	Population with systolic blood pressure 160 mm Hg or more.	1.8	80+	Incidence and mortality	Myocardial infarction and heart failure	Indapamide 1.5 mg	Placebo
MRC 2 Medical Research Council trial of treatment of hypertension	MRC Working Party, 1992 (ref. ^53^)	Hypertensive older patients without history of myocardial infarction or stroke, diabetes or impaired renal function within the preceding 3 months, had impaired renal function, asthma or any serious intercurrent disease	5.8	65–74	Incidence and mortality	Coronary heart disease	Diuretic or beta-blocker (atenolol 50 mg d^–1^ hydrochlorothiazide 25 or 50 mg d^–1^ + amiloride 2.5 or 5.0 mg d^–1^)	Placebo
MRFIT, Multiple Risk Factor Intervention Trial^a^	Stamler et al., 1989^a^ (ref. ^30^)	Men with no history of hospitalization for heart attack	6	35–57	Mortality	Coronary heart disease	NA^a^	NA^a^
NAVIGATOR	NAVIGATOR Study Group, 2010 (ref. ^54^)	Patients with impaired glucose tolerance, and one or more CVD risk factors or known CV disease	6.5	53–74	Incidence and mortality	Myocardial infarction, unstable angina and heart failure	Valsartan	Placebo
PART 2 The Prevention of Atherosclerosis with Ramipril trial	MacMahon et al., 200 (ref. ^51^)	Patients with hospital diagnosis (within 5 years of enrollment) or cardiovascular disease	4.7	49–75	Incidence and mortality	Coronary heart disease, myocardial infarction and unstable angina	Ramipril	Placebo
PATS Post-stroke Antihypertensive Treatment Study	Liu et al., 2009 (ref. ^47^)	Individuals with a history of stroke or transient ischemic attack	2	47–73	Incidence and mortality	Myocardial infarction	Indapamide 2.5 mg d^–1^	Placebo
PEACE, Prevention of Events with Angiotensin Converting Enzyme Inhibition Trial	Braunwald et al., 2004 (ref. ^37^)	Patients with stable coronary artery disease and normal or slightly reduced left ventricular function	4.8	52–76	Incidence	Myocardial infarction	Trandolapril 4 mg d^–1^	Placebo
PHARAO	Lüders et al., 2008 (ref. ^50^)	Internists and general practitioners with high-normal blood pressure	3	50–85	Incidence and mortality	Myocardial infarction	Ramipril 1.5 mg	Placebo
PREVEND IT	Asselbergs et al., 2004 (ref. ^35^)	Patients with angiographic evidence of coronary artery disease	3	30–80	Incidence and mortality	Myocardial infarction and angina	Fosinopril 20 mg	Placebo
PREVENT	Pitt et al., 2000 (ref. ^59^)	Patients with angiographic evidence of coronary artery disease	3	30–80	Incidence and mortality	Myocardial infarction and angina	Amlodipine	Placebo
PRoFESS Prevention Regimen For Effectively Avoiding Second Strokes Study	Yusuf et al., 2008 (ref. ^70^)	Patients who had had an ischemic stroke <90 days before randomization and whose condition was stable	3	55+	Incidence and mortality	Myocardial infarction	Telmisartan	Placebo
PROGRESS The perindopril protection against recurrent stroke study	PROGRESS Collaborative Group, 2001 (ref. ^61^)	Individuals with a history of stroke or transient ischemic attack	3.9	49–79	Incidence and mortality	Coronary heart disease	Perindopril 4 mg	Placebo
PSC, Prospective Studies Collaboration^a^	Lewington et al., 2002^a^ (ref. ^31^)	Adults with no previous vascular disease recorded at baseline		40–89	Mortality	IHD	NA	NA
RENAAL	Brenner et al., 2001 (ref. ^38^)	Patients with type 2 diabetes and nephropathy	3.4	31–70	Incidence and mortality	Myocardial infarction and heart failure	Losartan	Placebo
SCOPE, Study on COgnition and Prognosis in the Elderly	Lithell et al., 2003 (ref. ^45^)	Patients with mild to moderate hypertension	3.7	70–80	Incidence and mortality	Myocardial infarction	Candesartan 16 mg d^–1^	Placebo
SHEP Systolic Hypertension in the Elderly Program	SHEP Cooperative Research Group, 1984 (ref. ^63^)	Older population with isolated systolic hypertension	4.5	60+	Incidence and mortality	Coronary heart disease	For step 1 of the trial, dose 1 was chlorthalidone 12.5 mg d^–1^ or matching placebo; dose 2 was 25 mg d^–1^; for step 2, dose 1 was atenolol 25 mg d^–1^ or matching placebo; dose 2 was 50 mg/ d^–1^	Placebo
SPRINT	SPRINT Research Group, 2015 (ref. ^64^)	Individuals with systolic blood pressure 130–180 mm Hg and increased risk of CVD events	3.3	50+	Incidence and mortality	Myocardial infarction	Intensive treatment	Standard treatment
SPS3 Secondary Prevention of Small Subcortical Strokes trial	SPS3 Study Group, 2013 (ref. ^65^)	Individuals who had had a recent (within 180 days), symptomatic, agnetic resonance imaging-confirmed lacunar stroke and were without surgically amenable ipsilateral carotid artery stenosis or high-risk cardioembolic sources	3.7	30+	Incidence and mortality	Myocardial infarction	Lower target <130 mm Hg	Higher target (130–149 mm Hg)
STOP-Hypertension	Dahlöf et al., 1991 (ref. ^39^)	Untreated patients with systolic blood pressure 180 mm Hg or above or DBP >105 mm Hg, irrespective	2	70–82	Incidence and mortality	Myocardial infarction	Atenolol 50 mg, hydrochlorothiazide 25 mg + amiloride 2–5 mg, metoprolol 100 mg or pindolol 5 mg	Placebo
Syst-China	Liu et al., 1998 (ref. ^46^)	Older patients with isolated systolic hypertension and without cardiovascular disease	3	60+	Incidence and mortality	Coronary heart disease	Itrendipine, with the possible addition of captopril, hydrochlorothiazide or both	Placebo
The BBB Study	Hannson et al., 1994 (ref. ^42^)	Treated hypertensive patients with DBP 90–100 mm Hg and without history or clinical signs of coronary heart disease	5	45–67	Incidence and mortality	Myocardial infarction	Intensified treatment	Unchanged treatment to maintain DBP in the range 90–100 mm Hg
TOMHS	Neaton et al., 1993 (ref. ^55^)	Individuals not taking antihypertensive medication and with DBP 90–99 mm Hg.	4.4	45–69	Incidence and mortality	Coronary heart disease	Nutritional-hygienic intervention + one of the following: placebo; chlorthalidone 15 mg d^–1^; acebutolol 400 mg d^–1^; doxazosin mesylate 1 mg d^–1^ for 1 month, then 2 mg d^–1^; amlodipine maleate 5 mg d^–1^; or enalapril maleate 5 mg d^–1^	Placebo
TRANSCEND, Telmisartan Randomized Assessment Study	TRANSCEND Investigators, 2008 (ref. ^66^)	Angiotensin-converting enzyme (ACE)-intolerant subjects with cardiovascular disease	4.7	55+	Incidence and mortality	Myocardial infarction	Telmisartan 80 mg d^–1^	Placebo
UKPDS UK Prospective Diabetes Study (UKPDS 38)	UK Prospective Diabetes Study Group, 1999 (ref. ^68^)	Hypertensive patients with type 2 diabetes and without history of myocardial infarction in the previous year, current angina or heart failure	8.4	25+	Incidence and mortality	Myocardial infarction	ACE inhibitor to maximal doses or beta-blocker to maximal doses	Avoid ACE inhibitors and beta-blockers
VALISH Valsartan in Elderly Isolated Systolic Hypertension Study	Ogihara et al., 2010 (ref. ^57^)	Patients with isolated systolic hypertension	3.07	70–84	Incidence and mortality	Myocardial infarction	Valsartan	Valsartan

^a^Cohort studies. NA, not applicable.

The 39 RCTs included were identified in a systematic review conducted by Salam et al.^29^. Initially, 86 RCTs were selected from that review but 47 did not meet our inclusion criteria (specifically, 18 studies did not report IHD outcomes, 15 did not report baseline and/or follow-up SBP levels, 13 involved interventions that were irrelevant to this analysis or that raised SBP values and one study reported results in an unusable format). We additionally conducted a literature review of relevant RCTs published between February 2018 and April 2020 (Supplementary Information 2) that initially yielded 2,368 studies, with 109 selected for full-text screening; ultimately, none of these met our inclusion criteria (Methods and Extended Data Fig. 1).

Consistent with standard epidemiologic methods, the two cohort studies that we included—the Prospective Study Collaboration (PSC)^31^ and the Multiple Risk Factor Intervention Trial (MRFIT)^30^—excluded participants with a positive history or evidence at baseline of heart disease. In line with standard clinical trial approaches intended to examine subpopulations with increased risk, 37 out of the 39 RCTs included populations at increased risk of cardiovascular events; 12 included participants with cardiovascular disease history (including IHD, ischemic stroke, atrial fibrillation and heart failure); five included patients with type 2 diabetes; and two included patients with impaired renal function. Sixteen of the RCTs included hypertensive individuals, but only two of these studies included untreated hypertensive patients.

The mean age of participants across studies was 66.5 (s.d. = 12.5) years. For RCTs, mean follow-up was 3.9 years, ranging from 1.8 to 8.4 years. The cohort studies reported a mean follow-up of 6 years. Except for one study (MRFIT) that enrolled only men, all the above studies included men and women in similar proportions.

**Fig. 1:**
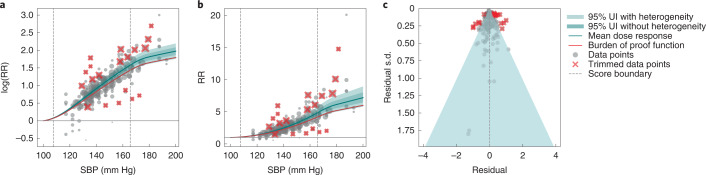
Relative risk of ischemic heart disease for different values of systolic blood pressure (SBP) in mm Hg. **a**, Log-relative risk (log(RR)) function. **b**, Relative risk (RR) function. **c**, Modified funnel plot showing the residuals (relative to 0) on the *x* axis and estimated s.d., including reported s.d. and between-study heterogeneity, on the *y* axis.

In the cohort studies, the lowest SBP category reported for a reference group was <115 mm Hg while the highest for a comparison group was >175 mm Hg. In RCTs, the observed average SBP ranged from 119.3 ± 10.1 to 166 ± 9.6 mm Hg in the reference/treatment groups and from 129 ± 10.3 to 192 ± 10.2 mm Hg in the comparison groups. Across all studies, only five studies included groups with SBP values <120 mm Hg at baseline, although the average of mean SBP exposure (defined as the midpoint of the lower and upper bounds of the exposure range/category associated with the reported risk measurement for the group in question) in the reference group was 121 mm Hg (100–166 mm Hg). The average mean SBP exposure in the alternate group was 146 mm Hg (115–193 mm Hg). The average mean SBP exposure among alternate and control groups was 135 mm Hg. We calculated the 15th percentile of exposure in the cohorts and trials to be an SBP of 107.5 mm Hg, and the 85th percentile to be 165 mm Hg (Fig. 1).

The clinical presentations of IHD reported in those studies included in this analysis were heterogeneous, primarily capturing acute IHD events such as myocardial infarction and new coronary heart disease (Table 2). The most extensively investigated IHD outcomes across studies were myocardial infarction (30 studies) and coronary heart disease (*n* = 16), followed by heart failure (*n* = 10), stable angina (*n* = 3), unstable angina (*n* = 2) and coronary revascularization (*n* = 2). Most studies (*n* = 38) reported the relative risk (RR) of IHD incidence and mortality combined, three studies reported IHD mortality only whereas two studies reported IHD incidence. The cohort studies provided RR estimates adjusted for age, sex, smoking and body mass index. All RCTs reported RRs that resulted from the intention-to-treat analysis. Most RCTs (*n* = 38) entailed control groups that had received placebos.

### Mean RR function

**Extended Data Fig. 3:**
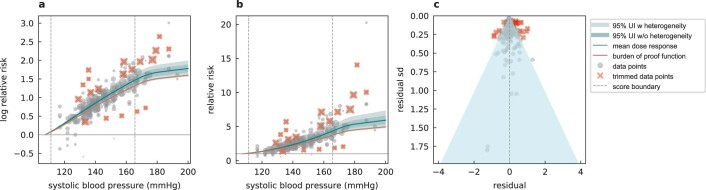
Relative risk of ischemic heart disease for different values of systolic blood pressure in mm Hg, starting at systolic blood pressure levels of 107.5 mm Hg. **a**, Log-relative risk (log(RR)) function. **b**, Relative risk (RR) function. **c**, Modified funnel plot showing the residuals (relative to 0) on the *x* axis and estimated s.d., including reported s.d. and between-study heterogeneity, on the *y* axis.

**Extended Data Fig. 4:**
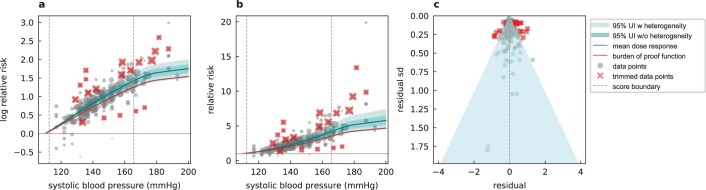
Relative risk of ischemic heart disease for different values of systolic blood pressure in mm Hg, starting at systolic blood pressure levels of 110 mm Hg. **a**, Log-relative risk (log(RR)) function. **b**, Relative risk (RR) function. **c**, Modified funnel plot showing the residuals (relative to 0) on the *x* axis and estimated s.d., including reported s.d. and between-study heterogeneity, on the *y* axis.

**Extended Data Fig. 5:**
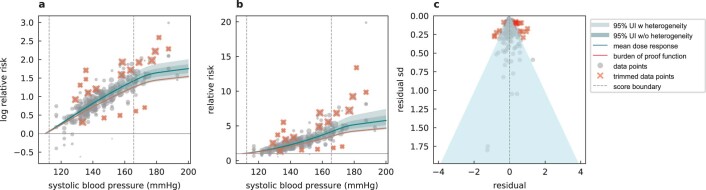
Relative risk of ischemic heart disease for different values of systolic blood pressure in mm Hg, starting at systolic blood pressure of 115 mm Hg. **a**, Log-relative risk (log(RR)) function. **b**, Relative risk (RR) function. **c**, Modified funnel plot showing the residuals (relative to 0) on the *x* axis and estimated s.d., including reported s.d. and between-study heterogeneity, on the *y* axis.

We found a significant harmful effect of elevated SBP levels on IHD. At the observed average SBP exposure of 135 mm Hg, the mean risk of IHD was 2.08 (95% UI inclusive of between-study heterogeneity = 1.91–2.26), compared with an SBP reference value of 100 mm Hg. We also established that modification of the presumed reference SBP values within the range <115 mm Hg did not substantially change the risk curve results. The mean RRs at 135 mm Hg relative to SBP values of 107.5, 110.0 and 115.0 were 2.17 (1.99–2.37), 2.11 (1.94–2.30) and 2.09 (1.93–2.23), respectively (Extended Data Figs. 3–5).

The mean RR function generated by our analytic approach showed a strong dose–response relationship, in which increasing SBP levels were associated with increased risk of IHD incidence and mortality across SBP values of 100–200 mm Hg (Fig. 1 and Supplementary Table 4). This risk curve is also available at http://vizhub.healthdata.org/burden-of-proof/. The risk function was nonlinear, flattening out at SBP values >165 mm Hg yet still increasing at higher SBP levels. Given the modeling strategy and data used for this analysis, in which plausible SBP values ranged 100–200 mm Hg, and considering that most of the available evidence reported IHD outcomes associated with values between the 15th and 85th percentile of the SBP exposure range (corresponding to 107.5–165.0 mm Hg), we examined the results stratified according to three different categories of SBP exposure: risk <107.5 mm Hg, risk 107.5–165 mm Hg and risk >165 mm Hg.

At SBP exposure values <107.5 mm Hg the available data were sparse and—even with extrapolation of the risk curve to SBP values 90–100 mm Hg and relaxing the monotonicity constraint—no evidence was observed of a J-shaped curve. We note that only IHD outcomes were included, so that risk due to non-IHD outcomes would not affect the risk curve. Below 107.5 mm Hg, the risk of IHD was null with a mean RR of 1.03 (1.00–1.03).

Within the segment of the RR curve over the exposure range 107.5–165.0 mm Hg, the association between SBP and IHD was approximately log-linear. Based on mean risk function, mean RR estimates for SBP levels of 110, 115, 120, 130, 140, 150 and 165 mm Hg were 1.12 (1.10–1.13), 1.23 (1.20–1.27), 1.39 (1.34–1.44), 1.81 (1.70–1.93), 2.38 (2.17–2.62), 3.11 (2.75–3.52) and 4.48 (3.81–5.26) compared to 100 mm Hg, respectively (Supplementary Table 4).

Above the 85th percentile of the mean SBP exposure range reported in the studies, corresponding to higher than 165 mm Hg, the mean RR was 5.72 (4.73–6.92). Although the data were sparse >165 mm Hg, the relationship between SBP and IHD had a flatter slope while still consistently increasing as it deviated from log-linearity.

### Between-study heterogeneity

Our analysis found consistent, but minor, between-study heterogeneity in the data (95% UIs inclusive of between-study heterogeneity represented by light green shading in Fig. 1a,b) after using a likelihood-based approach to trim 10% of the data (Fig. 1a,b) to ensure that the model fit the 90% most coherent data.

### Burden of proof risk function

Defined for harmful exposures as the 5th quantile of the risk curve closest to log(RR) = 0 or RR = 1 (that is, the null), BPRF represents a conservative estimate of the potential harmful effects of increased SBP on IHD based on the available evidence^24,25^, and is shown as a red curve in Fig. 1a,b.

Given the relatively minimal between-study heterogeneity observed in the data, our conservative estimate of the effect of SBP on IHD still showed a robust dose–response relationship, with an exposure-averaged BPRF of 2.01, indicating an increase of at least 101.36% in IHD risk across the range of SBP exposure 107.5–165.0 mm Hg relative to a reference value of 100 mm Hg. The BPRF yielded a ROS of 0.70 and a five-star rating, signifying that there was strong evidence supporting the statistically significant association between SBP and IHD (Supplementary Table 5).

Risk–outcome scores, star ratings, risk curves with all data points, trimmed data points and conventional and conservative uncertainty intervals and an interpretation of the findings are available for all risk–outcome pairs at http://vizhub.healthdata.org/burden-of-proof/.

### Systematic bias and publication bias

Using the bias covariate selection algorithm developed by Zheng et al.^24^, we found no systematic bias covariates that had a significant effect on RR function. With respect to publication or reporting bias, visual inspection of the symmetry of a customized funnel plot (Fig. 1c) does suggest potential risk of such bias among the studies included. This finding was supported by the statistically significant results of an Egger’s regression test^26^ with a *P* value of <0.05. It is likely that the use of pooled mean effect sizes resulted in the suggestion of bias in the funnel plot. Regardless, the magnitude of any such bias is small enough that it is unlikely to affect our main findings.

### Sensitivity analysis

**Extended Data Fig. 6:**
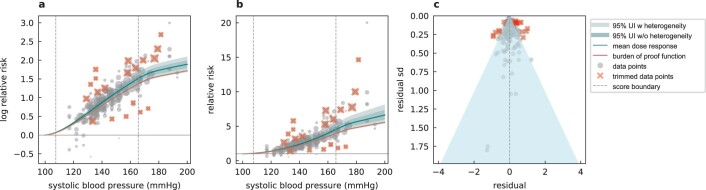
Relative risk of ischemic heart disease for different values of systolic blood pressure in mm Hg based, unconstrained. **a**, Log-relative risk (log(RR)) function. **b**, Relative risk (RR) function. **c**, Modified funnel plot showing the residuals (relative to 0) on the *x* axis and estimated s.d., including reported s.d. and between-study heterogeneity, on the *y* axis.

**Extended Data Fig. 7:**
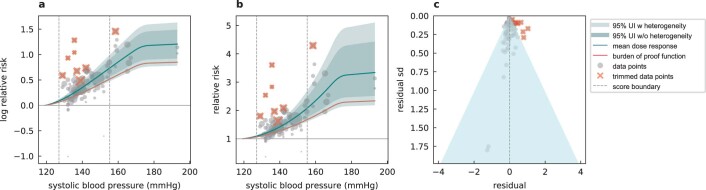
Relative risk of ischemic heart disease for different values of systolic blood pressure in mm Hg based on data from randomized control trials (RCTs). **a**, Log-relative risk (log(RR)) function. **b**, Relative risk (RR) function. **c**, Modified funnel plot showing the residuals (relative to 0) on the *x* axis and estimated s.d., including reported s.d. and between-study heterogeneity, on the *y* axis.

**Extended Data Fig. 8:**
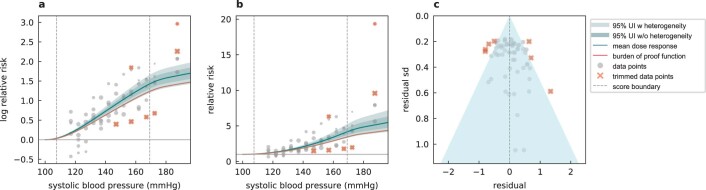
Relative risk of ischemic heart disease for different values of systolic blood pressure in mm Hg based on data from the Multiple Risk Factor Intervention Trial (MRFIT). **a**, Log-relative risk (log(RR)) function. **b**, Relative risk (RR) function. **c**, Modified funnel plot showing the residuals (relative to 0) on the *x* axis and estimated s.d., including reported s.d. and between-study heterogeneity, on the *y* axis.

**Extended Data Fig. 9:**
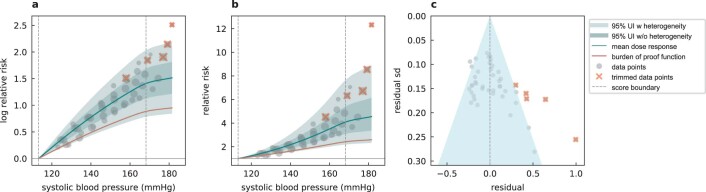
Relative risk of ischemic heart disease for different values of systolic blood pressure in mm Hg based on data from the Pacific Collaborator Research Pooled Cohort Study (PSC). **a**, Log-relative risk (log(RR)) function. **b**, Relative risk (RR) function. **c**, Modified funnel plot showing the residuals (relative to 0) on the *x* axis and estimated s.d., including reported s.d. and between-study heterogeneity, on the *y* axis.

**Extended Data Fig. 10:**
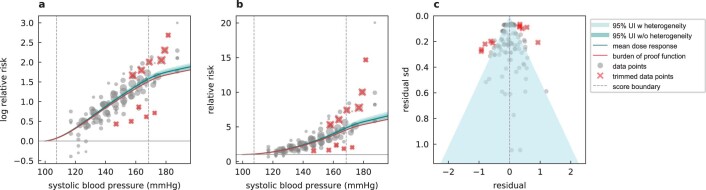
Relative risk of ischemic heart disease for different values of systolic blood pressure in mm Hg based on data from the Multiple Risk Factors Intervention Trial (MRFIT) and the Pacific Collaborator Research Pooled Cohort Study (PSC). **a**, Log-relative risk (log(RR)) function. **b**, Relative risk (RR) function. **c**, Modified funnel plot showing the residuals (relative to 0) on the *x* axis and estimated s.d., including reported s.d. and between-study heterogeneity, on the *y* axis.

We found that removal of the imposed model constraints related to linear tails and monotonicity did not have a notable effect on the shape of the risk curve or RR summary results (Extended Data Fig. 6). Further sensitivity analyses indicated that the study design—cohort studies versus RCTs—did not substantially influence the shape of the risk function (Extended Data Figs. 7–10). However, we observed that the uncertainty around estimates based only on data from RCTs was considerably larger than that based on data from the MRFIT trial and PSC study. Specifically, inclusion of data from the MRFIT study reduced the UI and modified BPRF and corresponding ROS. Based on a model including only RCT data, the BPRF value for IHD risk (averaged across BPRF between the 15th and 85th percentiles of exposure from the RCTs data, corresponding to SBP values of 127.0 and 155.27 mm Hg) was 1.33 based on an SBP reference value of 120 mm Hg, which corresponds to a ROS of 0.28 and a star rating of three. Using data solely from the MRFIT trial, we obtained an exposure-averaged BPRF of 1.81 based on an SBP reference exposure of 100 mm Hg, corresponding to a ROS of 0.59 and a star rating of four (in this case the 15th and 85th percentiles of exposure from the MRFIT data corresponded to SBP values of 107.5 and 169.2 mm Hg). Inclusion of data from only the PSC study resulted in a BPRF of 2.06 (based on an SBP reference exposure of 110 mm Hg and averaged between the 15th and 85th percentiles of SBP exposure, corresponding to values of 113.2 and 168.2 mm Hg), yielding a ROS of 0.72 and a five-star rating. In all cases, our conservative approach showed that SBP across the 15th–85th percentile exposure range increased the risk of IHD on average by at least 32%. Given that the cohort studies included a wider range of SBP values than the RCTs, and based on the similar risk function—in terms of both shape and magnitude, estimated for both cohorts and RCTs—we decided to include data from both cohort studies and RCTs.

## Discussion

Our results represent an updated systematic synthesis and meta-analysis of the available causal evidence from RCTs and cohort studies examining the effect of SBP on IHD outcomes. Broadly, the results showed a significant and direct dose–response relationship between SBP levels and IHD risk across all SBP exposure values (100–200 mm Hg), without evidence for a J-shaped curve. Based on mean risk function, SBP values of 130 and 140 mm Hg were associated with 81 and 138% higher risk of IHD, respectively, relative to the risk at 100 mm Hg, while an SBP of 165 mm Hg was associated with 348% higher risk. Similarly, when between-study heterogeneity was accounted for, the conservative BPRF interpretation of the evidence suggested that SBP values of 130, 140 and 165 mm Hg increased the risk of IHD by at least 76.8, 129.3 and 305.5%, respectively (relative to risk at 100 mm Hg).

Our data-driven, meta-regression approach allowed us not only to compute the mean risk function across different study designs and SBP exposure ranges^13^ by relaxing conventional log-linear assumptions, controlling for bias and explicitly handling differences in exposure ranges across studies—but also to generate BPRF, which quantifies and accounts for between-study heterogeneity. The BPRF provides (1) a rigorous approach to estimate the magnitude of the relationship between SBP and IHD, (2) a conservative interpretation of the available evidence and (3) a quantitative evaluation of evidence strength. The mean BPRF averaged across the data-dense range of SBP exposure between 107.5 and 165 mm Hg indicated that—even based on a highly conservative interpretation of the evidence—IHD risk increased on average by 101.36% over this range relative to an SBP of 100 mm Hg. The BPRF yielded a ROS of 0.70 and a five-star rating, indicative of a significant and robust dose–response association between SBP and IHD, supported by strong evidence.

While it is true that the relationship between SBP and IHD is well known, as established by previous meta-analyses^13,30,31,71^, the present findings contribute to the research and inform clinical practice and policy in a number of ways. Our results robustly confirm that IHD risk due to elevated SBP levels was high and evidence for the relationship strong. The statistical methods established by Zheng et al.^24,25^ resolve the longstanding reliance on assuming a log-linear relationship between SBP and IHD. Relaxing this assumption is particularly relevant to accurate estimation of the RR of SBP at its lowest and highest levels, which is where much of the scientific debate has focused. The ability afforded by our methodological framework to evaluate dose–response between exposure and outcome independent of any presumed statistical relationship—in addition to our approach that combined data from both RCTs and cohort studies to determine the shape of the risk function—supports our finding that there was no evidence of a J-shaped relationship between SBP and IHD at low SBP values. Additionally, the BPRF framework provides a standardized approach to quantify effect size and evidence strength, generating summary ROS and star-rating measures that simplify the communication and interpretation of the evidence. This will enable researchers, clinicians and policymakers to effectively evaluate and compare risks across other SBP-related outcomes as well as additional risk–outcome pairs.

Our findings also provide evidence that can inform some of the unanswered clinically relevant questions surrounding the relationship between SBP and IHD. Although resolution of inconsistencies between diagnostic SBP thresholds provided by disparate cardiovascular medical associations is beyond the scope of this paper—in particular, adjudication of whether SBP levels of 120–129 mm Hg should be considered normal or elevated and whether levels of 130–139 mm Hg are high-normal or hypertensive—the continuous risk function generated by our analysis indicates that relative IHD risk was already 1.39 (1.34–1.44) at 120 mm Hg SBP (relative to 100 mm Hg) and had risen to 1.81 (1.70–1.93) at 130 mm Hg, both SBP values that are considered ‘normal’ or low risk in some clinical guidelines^21,22^. Although the lack of data at both tails of the SBP exposure range limited us from making strong inferences at lower and higher SBP values, these findings suggest that (1) it is unlikely that reducing SBP at the low end of the exposure range can actually increase IHD risk^17–19^ and (2) increases in SBP level at the high end of the exposure range continue to raise IHD risk, albeit less steeply.

Overall, our findings call to the health community to prioritize the prevention and control of elevated SBP. Beyond clinical guidelines, a strategic approach for hypertension prevention, treatment and control is needed. While intervention trials suggest that lowering of SBP to levels <130 mm Hg with antihypertensive medications may be effective in reducing events only among selected higher-risk individuals, our results show that increased IHD risk should be expected at levels defined by certain current guidelines as normal or high-normal. This highlights the need to (1) enhance existing community screening programs to raise hypertension awareness and support timely diagnosis, (2) scale up effective treatment of hypertension to achieve universal coverage and (3) increase the technical capacity and resources allocated for primordial, primary and secondary hypertension and IHD prevention from early childhood through the life course.

Our approach has multiple benefits over traditional random effect models, including (1) allowing variable reference groups and exposure ranges that increase the use of available data and accuracy of the estimates, regardless of the shape of the underlying risk curve; (2) detecting outliers in the data based on the fit of the model; and (3) quantifying uncertainty due to between-study heterogeneity to evaluate evidence strength and generate a conservative interpretation of the available evidence. Moreover, our analysis incorporated evidence from both cohort studies and RCTs, allowing us to utilize a large amount of data both from participants with no history of or evidence at baseline of heart disease and those who were known to be at increased risk for cardiovascular events. Combining these different types of data—observations from the natural history of gradually increasing blood pressure versus the results of lowering blood pressure through pharmacotherapy—provides a broad synopsis of the available evidence on SBP–IHD risk but relies on the assumption of risk reversibility. Risk reversibility assumes that the risk of IHD that is accrued as blood pressure increases from level *x* to level *x* + *n* is equally informative as that eliminated by lowering blood pressure from level *x* + *n* back to level *x*. Our parallel sensitivity analyses of RCT and cohort studies show a very similar risk function, supporting the principle of risk reversibility on average for populations that may include individuals with and without prevalent IHD and its comorbidities. Such an assumption may be less valid for subpopulations with markedly different levels of underlying risk. For example, those with severe obstructive coronary artery disease may have a very different SBP–IHD risk function than that of the general population enrolled in cohorts and most RCTs focused on by our study, even though a small number of individuals in this general population are likely to have severe obstructive IHD.

Limitations of the present study include issues related to the input data and modeling approach. In terms of input data, potential limitations include (1) the low number of published studies that involved participants with either lower or extremely high SBP values; (2) lack of data on life course duration of elevated SBP before study enrollment in participants, which may impact the magnitude and shape of the IHD risk function; (3) variability among outcome definitions, including RR estimates for different combinations of the fatal and nonfatal endpoints of IHD; (4) lack of access to individual patient-level data, which would have supported more robust estimation; and (5) omission of relevant studies published after the 2020 cutoff date for our systematic literature review, such as Rahimi et al.^13^. In terms of the modeling approach, one potential limitation was that we chose to focus our analysis on SBP and did not evaluate IHD risk associated with isolated elevation in diastolic blood pressure (DBP). We made this choice because the two measurements are strongly correlated and because most studies report IHD risk solely in relation to SBP. Additionally, epidemiological studies have shown that SBP is a better predictor of health outcomes than DBP^12,30,72^. Other limitations associated with the modeling approach include (1) the inability to fully account for all potential factors or sources of heterogeneity and bias, regardless of the rigorous statistical methods used to limit confounding due to bias; (2) the absence of testing for bias that occurs when studies are more consistent with each other than expected by chance; and (3) the imposition of a right linear tail on the data, which assumes that increases in SBP >160 mm Hg are associated with smaller increments in IHD risk than are increases within the 110–165 mm Hg range. Combined with data sparsity for SBP levels >160 mm Hg, the use of a right linear tail constraint limits the inferences that can be made for populations with extremely high SBP exposure values. Similarly, at the other end of the SBP exposure range, components of the modeling approach that determined the minimum SBP value based on the available data and assumed the UI to be zero at this value restricted the ability to fully account for uncertainty in the exposure range where data were sparse.

The present study uses a meta-analytic approach^24^ to better understand the association between SBP and IHD. Our results suggest that IHD risk remains highest for individuals living with the highest SBP levels, providing evidence in support of public health and clinical interventions that will allow people to maintain SBP levels associated with low IHD risk throughout their lives.

## Methods

### Overview

This study was conducted as part of GBD 2020 (ref. ^2^). We used a meta-regression approach to analyze and interpret the available evidence to estimate the dose–response relationships between SBP and IHD, using a Bayesian regularized spline that captured the shape of the risk function from the data rather than imposing a log-linear relationship. We further improved accuracy by employing a robust, likelihood-based approach to detect and trim outliers, correcting for differences in exposure range across source studies and testing and controlling for bias related to study design. To complement the risk functions generated using this rigorous methodology, we also quantified between-study heterogeneity—a common source of bias in epidemiological studies—and used it to inform uncertainty.

From our uncertainty estimates we generated a BPRF representing the most conservative (that is, closest to null) interpretation of the severity of risk based on the available evidence and mapped the results onto a star rating system stratified into five levels of risk. The methodology and statistical techniques underlying this approach have been described previously^24,25^.

Among the six cardiovascular outcomes examined, IHD was selected to illustrate the case of a highly statistically significant risk–outcome relationship. Briefly, we (1) systematically gathered and reviewed the most relevant available evidence from published studies on the association between SBP and IHD; (2) estimated the shape of the risk–outcome relationship; (3) tested and adjusted for systematic bias; (4) quantified between-study heterogeneity; (5) evaluated publication and reporting bias; and (6) estimated BPRF to generate a conservative estimate of IHD risk across the SBP exposure range and assigned a star-rating risk category.

GBD 2020—under which the present study falls—used deidentified data, and the waiver of informed consent was reviewed and approved by the University of Washington Institutional Review Board (study no. 9060) up to, and including, 1 November 2022. This study complies with the Guidelines on Accurate and Transparent Health Estimate Reporting recommendations^73^ (Supplementary Table 6).

### Systematic review

We used a standardized approach to search for, and extract data from, published studies on the relationship between SBP and IHD. Building on the systematic review previously published by Salam et al.^29^, we initially screened the 86 studies included in that meta-analysis and additionally conducted an updated literature review of RCTs that compare the effect of blood pressure-lowering drugs versus placebos or compare different SBP targets. Databases were searched up to, and including, 1 April 2020 using keywords and medical subject headings for antihypertensive agents, blood pressure/drug effects and randomized trials published in English. The search string is fully detailed in Supplementary Information 2. Bibliographies of relevant publications were hand-searched to identify additional pertinent studies. Records were screened by reviewing titles and abstracts, and thereafter retrieved in full text.

Randomized control trials were included in the present analysis if they met the following criteria: (1) participants were randomly allocated to treatment versus control, or treatment target groups; (2) RR estimates (risk ratios, incidence rate ratios, odds ratios or hazard ratio) for incidence or mortality of an outcome of interest were reported for each group; (3) mean pre- and postintervention (or, alternatively, baseline and follow-up for cohort studies) SBP levels were reported for each group; and (4) outcomes of interest included myocardial infarction, angina, coronary heart disease, heart failure, major adverse cardiovascular events or revascularization cases. Studies were not excluded based on the presence or absence of any disease at baseline. Studies were excluded if they focused primarily on secondary hypertension or sudden cardiac death, severe arrhythmia, all-cardiovascular mortality or all-cause mortality. Head-to-head comparisons of different drug classes or trials of alternate blood pressure-lowering pharmacotherapies that were not intended to achieve identified target SBP levels were excluded. In addition to RCTs, we included data from large pooling projects for cohort studies previously used in GBD 2020 to estimate RR and the corresponding attributable burden of disease, and from cohort studies that have published the detailed level-specific RR needed for meta-analysis of this type. Specifically, results from the MRFIT^30^ observational study and the PSC pooled cohort^31^ were included in this meta-analysis.

Information on demographic characteristics, study design, sample size, follow-up duration, effect size and associated uncertainty, blood pressure levels and measurement methods, outcome definition, outcome ascertainment methods and number of IHD events was extracted. If the effect size and/or blood pressure levels were not reported directly, this information was estimated from published graphics of blood pressure over time or forest plots using the webplot digitizer application. For cohorts, covariates included in the statistical analysis of the study were also extracted. For RCTs, additional information on treatment and control groups was extracted. We extracted RRs specifically associated with the most highly specified diagnosis possible and excluded those of unspecified cardiovascular disease outcomes. IHD outcomes included in the analysis were coronary artery disease, heart failure, stable angina, unstable angina and coronary revascularization. Supplementary Table 7 presents the categories of data extracted from the studies during the systematic literature review (Extended Data Fig. 2).

The estimates we generated—RR, BPRF, ROS and star rating—are neither specific to nor disaggregated by specific populations, including by sex or gender. We included all available data regardless of how or whether the input study collected and reported data by sex or gender. From the 41 studies included in the study, 100% included information about the self-reported sex of the participants but none reported IHD RR estimates by sex, precluding us from performing any sex- or gender-based analyses.

### Estimating the shape of the risk–outcome relationship

To ensure that we characterized the functional form of the association between SBP and IHD as accurately as possible, we avoided the conventional assumption of a log-linear dose–response relationship, using Bayesian regularized spline meta-regression with the RR of IHD modeled as the dependent variable and SBP exposure values in mm Hg as a continuous independent variable. An initial model was run with no priors or constraints imposed on the shape of the relationship and no covariates included. Based on the results of this exploratory analysis—in which the RR curve increased and remained >1 over the entire SBP exposure domain and no evidence of a J-shaped risk curve was found—we elected to incorporate statistical priors and constraints to strengthen the findings based on the data. We fit a final model consisting of a nonlinear dose–response with a monotonicity constraint imposed across the SBP exposure range and a linear tail constraint on the right side of the exposure domain to ensure plausible risk curve behavior at high and low exposure levels. Specifically, we used a quadratic spline with two interior knots, linear tails and a prior on the maximum derivative of the right linear tail (mean = 0, s.d. = 0.001). To make our results robust to knot placement, an ensemble model was created from 50 models using random knot placement by optimizing for model fit (based on a likelihood metric) and total variation (based on the highest derivative). To further improve the accuracy of our results, outliers were identified and removed as part of the model-fitting process using a likelihood-based approach that trimmed 10% of the data, ensuring that the model fit the 90% most coherent data. Moreover, because our analysis relaxed the assumption of a log-linear relationship between SBP and IHD and, because most of the input data came from RCTs comparing groups with different reference and exposure ranges, we explicitly handled these differences by integrating the risk functions over exposure ranges and including this mechanism in the likelihood.

### Testing and adjusting for bias related to study attributes

We quantified study attributes that could potentially bias the effect size estimates creating three types of dummy bias covariates (*αX* covariates that explain variation in the true effect, *βχ* that predict bias in measurement and *γZ* that explain differences in between-study heterogeneity). Overall, covariates captured information related to different definitions of the outcome (angina, IHD, coronary heart disease, revascularization), study type (RCT or cohort), use of RRs versus odds ratios or hazard ratios to quantify effect size, reliance on measures of incidence versus mortality, use of single or repeat SBP measurements, outcome determination based on administrative records or self-report and, for cohort studies, the extent of adjustment for relevant confounders such as age, sex, smoking, education, body mass index, cholesterol measurements and income. For RCTs, covariates capturing whether placebo versus a different intervention were used, and adherence to treatment, follow-up and randomization and blinding were also assessed. Detailed definitions of the bias covariates assessed in the analysis are presented in Supplementary Table 8. We then followed the approach of Zheng and colleagues^24,25^ to systematically test for potential bias covariates that had a significant effect on the risk–outcome function (corrected for number of studies used); we ranked covariates using a Lasso covariate selection scheme to acquire ordering of the most impactful to least and added them one at a time, based on their ranking, to a linear meta-regression modeling the effect of SBP on IHD. The precision of the relative sizes of data points is directly included in the optimization formulation used to construct the Lasso approach^24^—and so affects both the ordering of covariates and the final set of bias covariates. We then adjusted for any significant bias covariates in the final meta-regression analysis.

### Quantification of between-study heterogeneity

To capture between-study heterogeneity—that is, disparities across the estimates obtained from the input sources for the meta-analysis—we entered the mean risk–outcome function and selected bias covariates obtained from our previous steps into a linear mixed-effects model that scaled the RR yielded by each study using study-specific random slopes. We estimated the variance of between-study random effects using the Fisher information matrix, which is robust to both data sparsity and the presence of within-study correlation. We report our main estimates of mean RR using uncertainty intervals that include the effect of between-study heterogeneity, from which we derived BPRF and associated ROS and star rating.

### Evaluation of publication and reporting bias

We evaluated publication and reporting bias based on visual inspection of the funnel plot, which shows the degree to which the mean effect size is correlated with s.d., and on statistical testing of this relationship using the classic Egger’s regression^26^ strategy applied to the residuals of the model (Fig. 1). Our approach did not find any indication of publication or reporting bias in the studies included in our meta-analyses.

### BPRF and star-rating system

We used the evidence score framework established in GBD 2020 to systematically estimate the risk function representing the relationship between SPB and IHD, and to quantify the strength of evidence for the estimated relationship. Using uncertainty estimates computed to include between-study heterogeneity we derived BPRF, defined in this case (for harmful risks) as the 5th quantile risk curve (closest to an RR of 1 representing the null). For the relationship between SBP and IHD, BPRF represents the estimated level of elevated IHD risk based on a conservative interpretation of the available evidence—that is, the minimum estimate of the potential harmful effects of increased SBP on IHD. We acknowledge that the quantile depends closely on the risk factor range and therefore we defined this range based on RCTs and cohort exposure data.

To summarize BPRF in a single measure and to be able to compare the strength of the evidence across risk–outcome pairs of varying nature, we then generated a ROS from the average log(RR) of BPRF over the data-dense area of the observed exposure range, which we defined as the 15th–85th percentiles of SBP exposure. A higher positive ROS corresponds to a higher average effect size across the continuum of risk, and stronger evidence for the estimated relationship. To provide easy interpretation of ROS, and for comparative purposes, we categorized the ROS value using a star-rating system from one to five; negative ROSs yield a one-star ranking and indicate risks for which the mean relationship is statistically significant as conventionally assessed (based on uncertainty estimates exclusive of between-study heterogeneity), but is not significant based on our conservative analysis of the available evidence, suggesting there may be no true association between risk exposure and health outcome. Positive ROS ranges were divided as follows: two stars represent at least a 0–15% risk increase based on average SBP exposure, three stars indicate >15–50% increase in risk, four stars >50–85% increase and five stars >85% increase. Translated into ROS values, the five-star-rating ranges are <0.0, 0.0–0.14, >0.14–0.41, >0.41–0.62 and >0.62.

Subgroup and sensitivity analyses were conducted to clarify whether the results were consistent across study characteristics (for example, sex, duration of follow-up, geographic location, study quality, study outcome definition and adjustment for confounding factors), and to ensure that they were not driven by large studies, extreme results or SBP exposure range.

The validity of the approach used here to meta-analyze the data has been extensively evaluated by Zheng et al.^2,24,25^. For this analysis, dose–response risk estimates were validated by plotting the mean risk function along with its 95% uncertainty interval against both the extracted dose-specific RR data from the studies included and dose–response risk estimates from the GBD 2019 study^2^. Mean risk functions, along with 95% UIs, were validated based on the fit of the data, the shape of the relationship and the plausibility of dose–response risk curves. As a final step, risk curves were validated and approved by all authors.

### Statistical analysis

Data were extracted and prepared in Microsoft Excel and analyzed using comprehensive meta-analysis software. Analyses were carried out using R v.3.6.2 and Python v.3.8.

### Statistics and reproducibility

The study was a secondary analysis of existing data involving systematic reviews and meta-analyses. No statistical method was used to predetermine sample size. Because the study did not involve primary data collection, randomization, blinding and data exclusions are not relevant, and because such no data were excluded and we performed no randomization or blinding. We have made our data and code available, to foster reproducibility.

### Reporting summary

Further information on research design is available in the Nature Research Reporting Summary linked to this article.

## Online content

Any methods, additional references, Nature Research reporting summaries, source data, extended data, supplementary information, acknowledgements, peer review information; details of author contributions and competing interests; and statements of data and code availability are available at 10.1038/s41591-022-01974-1.

## Supplementary information

Supplementary Information Supplementary Information

Reporting Summary

## Data Availability

The findings from this study are supported by data available in public online repositories and data that are available upon request from the data provider; nonpublicly available data were used under license for the current study, but can be made available with permission of the data provider; contact information is provided where applicable. Data sources and citations for each risk–outcome pair can be downloaded using the ‘download’ button on each risk curve page currently available at http://vizhub.healthdata.org/burden-of-proof. See Supplementary Table 3 for a list of studies from which we extracted data for use in our analysis, along with relevant study characteristics. Citations for all the studies used can be found in Systematic review and are also provided, alphabetized according to study name or acronym, in Table 2.

## Code availability

All code used for these analyses is publicly available online (https://github.com/ihmeuw-msca/burden-of-proof). This includes code for the meta-regression engine, the model specification interface, both parts of the data processing and risk-specific custom code, as appropriate. To validate key aspects of the meta-regression model used in this analysis, the following packages were used: metafor (R package available for download at 10.18637/jss.v036.i03) and dosmesreta (R package available for download at https://www.jstatsoft.org/article/view/v072c01).
