# Supplementary material for: Effects of elevated systolic blood pressure on ischemic heart disease: a Burden of Proof study
Source: Nat Med. 2022 Oct 10;28(10):2056–65. doi: 10.1038/s41591-022-01974-1 (PMC9556328; doi:10.1038/s41591-022-01974-1)
Supplement: Supplementary file 1 — Supplementary Information [file 41591_2022_1974_MOESM1_ESM.pdf]

nature portfolio

https://doi.org/10.1038/s41591-022-01974-1

Supplementary information

Effects of elevated systolic blood pressure on ischemic heart disease: a Burden of Proof study

In the format provided by the authors and unedited

Supplementary Information: data sources and supplementary results for “Effects of elevated systolic blood pressure on ischemic heart disease: a Burden of Proof study"

This appendix provides detailed information on input data sources and supplementary results for “Effects of elevated systolic blood pressure on ischemic heart disease: a Burden of Proof study."

Table of Contents

Section 1. Supplementary tables *2*

Supplementary Table 1. PRISMA 2020 checklist 2

Supplementary Table 2. PRISMA 2020 abstract checklist 5

Supplementary Table 3. Study name and citation for all input data sources 5

Supplementary Table 4. Mean relative risk measures of IHD risk across systolic blood pressure exposure 8

Supplementary Table 5. Burden of proof risk function for high systolic blood pressure exposure and ischemic heart disease. 9

Supplementary Table 6. GATHER checklist 9

Supplementary Table 7. Causal criteria extraction template 10

Supplementary Table 8. Study covariates assessed in the analysis 16

Section 2: Data source identification *16*

Section 2.1 Literature studies 17

Section 2.1.1 PubMed search 17

Section 3: Sensitivity analysis *18*

Section 3.1. Model results based on input data: testing cohort studies vs RCTs 18

Section 1. Supplementary tables

Supplementary Table 1. PRISMA 2020 checklist

Section and Topic	Item #	Checklist item	Location where item is reported
**TITLE**			
Title	1	Identify the report as a systematic review.	This study leveraged a review of the literature as described in the Methods section, "systematic review".
**ABSTRACT**			
Abstract	2	See the PRISMA 2020 for Abstracts checklist.	See PRISMA 2020 for Abstracts Checklist (Supplementary Table 2)
**INTRODUCTION**			
Rationale	3	Describe the rationale for the review in the context of existing knowledge.	"Main" (intro) paragraphs 1-3
Objectives	4	Provide an explicit statement of the objective(s) or question(s) the review addresses.	"Main" (intro) paragraph 5
**METHODS**			
Eligibility criteria	5	Specify the inclusion and exclusion criteria for the review and how studies were grouped for the syntheses.	Full inclusion and exclusion criteria listed in Methods section "literature review"; reasons for exclusion and number of studies excluded also provided in PRISMA flow diagram (Extended Data Figure 1)
Information sources	6	Specify all databases, registers, websites, organisations, reference lists and other sources searched or consulted to identify studies. Specify the date when each source was last searched or consulted.	Methods section "systematic review"; Supplementary Information Section 2
Search strategy	7	Present the full search strategies for all databases, registers and websites, including any filters and limits used.	Methods section "literature review"; Supplementary Information Section 2.1
Selection process	8	Specify the methods used to decide whether a study met the inclusion criteria of the review, including how many reviewers screened each record and each report retrieved, whether they worked independently, and if applicable, details of automation tools used in the process.	Methods section "systematic review"
Data collection process	9	Specify the methods used to collect data from reports, including how many reviewers collected data from each report, whether they worked independently, any processes for obtaining or confirming data from study investigators, and if applicable, details of automation tools used in the process.	Methods section "systematic review"
Data items	10a	List and define all outcomes for which data were sought. Specify whether all results that were compatible with each outcome domain in each study were sought (e.g. for all measures, time points, analyses), and if not, the methods used to decide which results to collect.	Title, abstract, methods sections
	10b	List and define all other variables for which data were sought (e.g. participant and intervention characteristics, funding sources). Describe any assumptions made about any missing or unclear information.	Methods section "systematic review"; Results section table 2 "Study characteristics" for each included study full list and definitions of all variables are in Supplementary Information Table 7
Study risk of bias assessment	11	Specify the methods used to assess risk of bias in the included studies, including details of the tool(s) used, how many reviewers assessed each study and whether they worked independently, and if applicable, details of automation tools used in the process.	Overview of methods for testing for bias in main text methods section "testing for bias across different study designs and characteristics"
Effect measures	12	Specify for each outcome the effect measure(s) (e.g. risk ratio, mean difference) used in the synthesis or presentation of results.	Main methods "overview" and "estimating the burden of proof risk function" sections.

Synthesis methods	13a	Describe the processes used to decide which studies were eligible for each synthesis (e.g. tabulating the study intervention characteristics and comparing against the planned groups for each synthesis (item #5)).	Broad description of processes available in methods “literature reviews”
	13b	Describe any methods required to prepare the data for presentation or synthesis, such as handling of missing summary statistics, or data conversions.	Methods section “literature review”
	13c	Describe any methods used to tabulate or visually display results of individual studies and syntheses.	Methods sections “literature reviews”, “estimating the shape of the risk-outcome relationship”; Figure 1
	13d	Describe any methods used to synthesize results and provide a rationale for the choice(s). If meta-analysis was performed, describe the model(s), method(s) to identify the presence and extent of statistical heterogeneity, and software package(s) used.	Methods sections “Estimating the shape of the risk-outcome relationship,” “quantifying between-study heterogeneity...,” “estimating the burden of proof risk function”. Software packages described in “code availability” section of the manuscript
	13e	Describe any methods used to explore possible causes of heterogeneity among study results (e.g. subgroup analysis, meta-regression).	Methods section “quantifying between-study heterogeneity” and results of sensitivity analyses Extended Data Figures 3-10
	13f	Describe any sensitivity analyses conducted to assess robustness of the synthesized results.	Supplementary Information Section 3: sensitivity results (reference to these results found in the main text of the methods and results sections “sensitivity analysis” and Extended Data Figures 3-10
Reporting bias assessment	14	Describe any methods used to assess risk of bias due to missing results in a synthesis (arising from reporting biases).	Methods for detecting publication or reporting bias found in methods section “evaluating publication and reporting bias”
Certainty assessment	15	Describe any methods used to assess certainty (or confidence) in the body of evidence for an outcome.	Methods section “quantifying between-study heterogeneity”
**RESULTS**			
Study selection	16a	Describe the results of the search and selection process, from the number of records identified in the search to the number of studies included in the review, ideally using a flow diagram.	PRISMA flow diagram Extended Data Figure 1
	16b	Cite studies that might appear to meet the inclusion criteria, but which were excluded, and explain why they were excluded.	Not applicable
Study characteristics	17	Cite each included study and present its characteristics.	Results Table 2 (“study characteristics”); Supplemental Information Table S3 (“study name and citation for all input data sources”) citations also provided in the online viz tool.
Risk of bias in studies	18	Present assessments of risk of bias for each included study.	Results section “burden of proof risk function”
Results of individual studies	19	For all outcomes, present, for each study: (a) summary statistics for each group (where appropriate) and (b) an effect estimates and its precision (e.g. confidence/credible interval), ideally using structured tables or plots.	No, we do not present this information in the present manuscript.
Results of syntheses	20a	For each synthesis, briefly summarise the characteristics and risk of bias among contributing studies.	Results section “burden of proof risk function” and methods section “testing and adjusting for bias related to study attributes”
	20b	Present results of all statistical syntheses conducted. If meta-analysis was done, present for each the summary estimate and its precision (e.g. confidence/credible interval) and	Results, Figure 1; Supplemental Information Table 4 and 5; Extended

		measures of statistical heterogeneity. If comparing groups, describe the direction of the effect.	Data Figures 3-10 (“relative risks across exposure range”)
	20c	Present results of all investigations of possible causes of heterogeneity among study results.	All uncertainty intervals presented everywhere in the manuscript and appendices reflect between-study heterogeneity (unless specified otherwise); BPRFs, ROSs, and star-ratings for each risk-outcome pair also reflect between-study heterogeneity
	20d	Present results of all sensitivity analyses conducted to assess the robustness of the synthesized results.	Supplemental Information Section 3 (reference to these results given in Results “Sensitivity analysis” section) and Extended Data Figures 3-10
Reporting biases	21	Present assessments of risk of bias due to missing results (arising from reporting biases) for each synthesis assessed.	Results section “systematic bias and publication bias;” funnel plots (figure 1); Extended data figures 6-10 for sensitivity analysis results
Certainty of evidence	22	Present assessments of certainty (or confidence) in the body of evidence for each outcome assessed.	All estimates are presented with 95% uncertainty intervals. UI values are given alongside all mean estimates in the Results and Discussion sections as well as in Supplementary Table S4 and S5; all risk curve figures (Figure 1; Extended Data Figures 3-10) include shading to depict UI curves (both with and without between-study heterogeneity)
**DISCUSSION**			
Discussion	23a	Provide a general interpretation of the results in the context of other evidence.	Discussion paragraphs 4, 5 & 6
	23b	Discuss any limitations of the evidence included in the review.	Discussion paragraph 8
	23c	Discuss any limitations of the review processes used.	Discussion paragraph 8
	23d	Discuss implications of the results for practice, policy, and future research.	Discussion paragraph 4
**OTHER INFORMATION**			
Registration and protocol	24a	Provide registration information for the review, including register name and registration number, or state that the review was not registered.	This protocol is part of the Global Burden of Diseases, Injuries, and Risk Factors Study which has been registered and approved through the University of Washington Institutional Review Board as described in the Methods overview section. The literature review was not registered on its own.
	24b	Indicate where the review protocol can be accessed, or state that a protocol was not prepared.	No
	24c	Describe and explain any amendments to information provided at registration or in the protocol.	No
Support	25	Describe sources of financial or non-financial support for the review, and the role of the funders or sponsors in the review.	“Acknowledgments” section of the manuscript
Competing interests	26	Declare any competing interests of review authors.	“Competing interests” section of the manuscript

Availability of data, code and other materials	27	Report which of the following are publicly available and where they can be found template data collection forms; data extracted from included studies; data used for all analyses; analytic code; any other materials used in the review.	"Data availability" and "code availability" sections in the manuscript; data collection form template: Supplemental Information Table S47

Supplementary Table 2. PRISMA 2020 abstract checklist

Section and Topic	Item #	Checklist item	Reported (Yes/No)
**TITLE**			
Title	1	Identify the report as a systematic review.	Not applicable
**BACKGROUND**			
Objectives	2	Provide an explicit statement of the main objective(s) or question(s) the review addresses.	Yes
**METHODS**			
Eligibility criteria	3	Specify the inclusion and exclusion criteria for the review.	Yes, in the main text
Information sources	4	Specify the information sources (e.g. databases, registers) used to identify studies and the date when each was last searched.	Yes, in the main text
Risk of bias	5	Specify the methods used to assess risk of bias in the included studies.	Yes, in the main text
Synthesis of results	6	Specify the methods used to present and synthesise results.	Yes
**RESULTS**			
Included studies	7	Give the total number of included studies and participants and summarise relevant characteristics of studies.	Yes, in the main text and Supplementary Information
Synthesis of results	8	Present results for main outcomes, preferably indicating the number of included studies and participants for each. If meta-analysis was done, report the summary estimate and confidence/credible interval. If comparing groups, indicate the direction of the effect (i.e. which group is favoured).	Yes, the number of included studies and participants is reported in the main text
**DISCUSSION**			
Limitations of evidence	9	Provide a brief summary of the limitations of the evidence included in the review (e.g. study risk of bias, inconsistency and imprecision).	Yes, in the main text
Interpretation	10	Provide a general interpretation of the results and important implications.	Yes
**OTHER**			
Funding	11	Specify the primary source of funding for the review.	Yes, in the main text
Registration	12	Provide the register name and registration number.	No

Supplementary Table 3. Study name and citation for all input data sources

Study name	Citation
ABCD-N	Schrier, R. W., Estacio, R. O., Esler, A. & Mehler, P. Effects of aggressive blood pressure control in normotensive type 2 diabetic patients on albuminuria, retinopathy and strokes. Kidney Int 61, 1086–1097 (2002).

Study name	Citation
ACCORD, Action to Control Cardiovascular Risk in Diabetes Study	ACCORD Study Group et al. Effects of intensive blood-pressure control in type 2 diabetes mellitus. N Engl J Med 362, 1575–1585 (2010).
ACTION Trial	Poole-Wilson, P. A. *et al.* Effect of long-acting nifedipine on mortality and cardiovascular morbidity in patients with stable angina requiring treatment (ACTION trial): randomised controlled trial. *Lancet* **364**, 849–857 (2004).
Active I	ACTIVE I Investigators *et al.* Irbesartan in patients with atrial fibrillation. *N Engl J Med* **364**, 928–938 (2011).
ADVANCE	Patel, A. et al. Effects of a fixed combination of perindopril and indapamide on macrovascular and microvascular outcomes in patients with type 2 diabetes mellitus (the ADVANCE trial): a randomised controlled trial. Lancet 370, 829–840 (2007).
CAMELOT	Nissen, S. E. *et al.* Effect of antihypertensive agents on cardiovascular events in patients with coronary disease and normal blood pressure: the CAMELOT study: a randomized controlled trial. *JAMA* **292**, 2217–2225 (2004).
CARDIO-SIS	Verdecchia, P. *et al.* Usual versus tight control of systolic blood pressure in non-diabetic patients with hypertension (Cardio-Sis): an open-label randomised trial. *Lancet* **374**, 525–533 (2009).
DIABHYCAR	Marre, M. *et al.* Effects of low dose ramipril on cardiovascular and renal outcomes in patients with type 2 diabetes and raised excretion of urinary albumin: randomised, double blind, placebo controlled trial (the DIABHYCAR study). *BMJ* **328**, 495 (2004).
DREAM, Diabetes Reduction Assessment with ramipril and rosiglitazone Medication	DREAM Trial Investigators *et al.* Effect of ramipril on the incidence of diabetes. *N Engl J Med* **355**, 1551–1562 (2006).
Dutch TIA	The Dutch TIA Trial Study Group. Trial of secondary prevention with atenolol after transient ischemic attack or nondisabling ischemic stroke. *Stroke* **24**, 543–548 (1993)
EUROPA, European trial on Reduction of cardiac events with Perindopril in patients with stable coronary artery disease study	Fox, K. M. & EURopean trial On reduction of cardiac events with Perindopril in stable coronary Artery disease Investigators. Efficacy of perindopril in reduction of cardiovascular events among patients with stable coronary artery disease: randomised, double-blind, placebo-controlled, multicentre trial (the EUROPA study). *Lancet* **362**, 782–788 (2003).
EWPHE, European Working Party on High Blood Pressure in the Elderly	Amery, A. *et al.* Mortality and morbidity results from the European Working Party on High Blood Pressure in the Elderly trial. *Lancet* **1**, 1349–1354 (1985).
FEVER, Felodipine Event Reduction Study	Liu, L. *et al.* The Felodipine Event Reduction (FEVER) Study: a randomized long-term placebo-controlled trial in Chinese hypertensive patients. *J Hypertens* **23**, 2157–2172 (2005).
HOPE-3, Heart Outcomes Prevention Evaluation study 3	Lonn, E. M. *et al.* Blood-pressure lowering in intermediate-risk persons without cardiovascular disease. *N Engl J Med* **374**, 2009–2020 (2016).
HOPE, Heart Outcomes Prevention Evaluation study	Heart Outcomes Prevention Evaluation Study Investigators *et al.* Effects of an angiotensin-converting-enzyme inhibitor, ramipril, on cardiovascular events in high-risk patients. *N Engl J Med* **342**, 145–153 (2000).
HOT, Hypertension Optimal Treatment	Hansson, L. *et al.* Effects of intensive blood-pressure lowering and low-dose aspirin in patients with hypertension: principal results of the Hypertension Optimal Treatment (HOT) randomised trial. HOT Study Group. *Lancet* **351**, 1755–1762 (1998).
HYVET, Hypertension in the Very Elderly Trial	Beckett, N. S. *et al.* Treatment of hypertension in patients 80 years of age or older. *N Engl J Med* **358**, 1887–1898 (2008).

Study name	Citation
MRC 2, Medical Research Council trial of treatment of hypertension	MRC Working Party. Medical Research Council trial of treatment of hypertension in older adults: principal results. *BMJ* **304**, 405–412 (1992).
MRFIT, Multiple Risk Factor Intervention Trial	Stamler, J., Neaton, J. D. & Wentworth, D. N. Blood pressure (systolic and diastolic) and risk of fatal coronary heart disease. *Hypertension* **13**, I2 (1989).
NAVIGATOR	NAVIGATOR Study Group *et al.* Effect of valsartan on the incidence of diabetes and cardiovascular events. *N Engl J Med* **362**, 1477–1490 (2010).
PART 2 The Prevention of Atherosclerosis with Ramipril trial	MacMahon, S. *et al.* Randomized, placebo-controlled trial of the angiotensin-converting enzyme inhibitor, ramipril, in patients with coronary or other occlusive arterial disease. PART-2 Collaborative Research Group. Prevention of Atherosclerosis with Ramipril. *J Am Coll Cardiol* **36**, 438–443 (2000).
PATS, Post-stroke Antihypertensive Treatment Study	Liu, L. *et al.* Blood pressure reduction for the secondary prevention of stroke: a Chinese trial and a systematic review of the literature. *Hypertens Res* **32**, 1032–1040 (2009).
PEACE, Prevention of Events with Angiotensin Converting Enzyme Inhibition Trial	Braunwald, E. *et al.* Angiotensin-converting-enzyme inhibition in stable coronary artery disease. *N Engl J Med* **351**, 2058–2068 (2004).
PHARAO	Lüders, S. *et al.* The PHARAO study: prevention of hypertension with the angiotensin-converting enzyme inhibitor ramipril in patients with high-normal blood pressure: a prospective, randomized, controlled prevention trial of the German Hypertension League. *J Hypertens* **26**, 1487–1496 (2008).
PREVEND IT	Asselbergs, F. W. *et al.* Effects of fosinopril and pravastatin on cardiovascular events in subjects with microalbuminuria. *Circulation* **110**, 2809–2816 (2004).
PREVENT	Pitt, B. *et al.* Effect of amlodipine on the progression of atherosclerosis and the occurrence of clinical events. PREVENT Investigators. *Circulation* **102**, 1503–1510 (2000).
PRoFESS, Prevention Regimen for Effectively Avoiding Second Strokes Study	Yusuf, S. *et al.* Telmisartan to prevent recurrent stroke and cardiovascular events. *N Engl J Med* **359**, 1225–1237 (2008).
PROGRESS, The perindopril protection against recurrent stroke study	PROGRESS Collaborative Group. Randomised trial of a perindopril-based blood-pressure-lowering regimen among 6,105 individuals with previous stroke or transient ischaemic attack. *Lancet* **358**, 1033–1041 (2001).
PSC, Prospective Studies Collaboration	Lewington, S. *et al.* Age-specific relevance of usual blood pressure to vascular mortality: a meta-analysis of individual data for one million adults in 61 prospective studies. *The Lancet* **360**, 1903–1913 (2002).
QUIET, Quinapril Ischemic Event Trial	Pitt, B. *et al.* The QUinapril Ischemic Event Trial (QUIET): evaluation of chronic ACE inhibitor therapy in patients with ischemic heart disease and preserved left ventricular function. *Am J Cardiol* **87**, 1058–1063 (2001).
RENAAL, The Reduction of Endpoints in NIDDM with the Angiotensin II Antagonist Losartan Study	Brenner, B. M. *et al.* Effects of losartan on renal and cardiovascular outcomes in patients with type 2 diabetes and nephropathy. *N Engl J Med* **345**, 861–869 (2001).
SCOPE, Study on Cognition and Prognosis in the Elderly	Lithell, H. *et al.* The Study on Cognition and Prognosis in the Elderly (SCOPE): principal results of a randomized double-blind intervention trial. *J Hypertens* **21**, 875–886 (2003).
SHEP, Systolic Hypertension in the Elderly Program	SHEP Cooperative Research Group. Prevention of stroke by antihypertensive drug treatment in older persons with isolated systolic hypertension. Final results of the Systolic Hypertension in the Elderly Program (SHEP). *JAMA* **265**, 3255–3264 (1991).
SPRINT	SPRINT Research Group. A randomized trial of intensive versus standard blood-pressure control. *New England Journal of Medicine* **373**, 2103–2116 (2015).

Study name	Citation
SPS3, Secondary Prevention of Small Subcortical Strokes trial	SPS3 Study Group *et al.* Blood-pressure targets in patients with recent lacunar stroke: the SPS3 randomised trial. *Lancet* **382**, 507–515 (2013).
STOP-Hypertension	Dahlöf, B. *et al.* Morbidity and mortality in the Swedish Trial in Old Patients with Hypertension (STOP-Hypertension). *Lancet* **338**, 1281–1285 (1991).
Syst-China	Liu, L., Wang, J. G., Gong, L., Liu, G. & Staessen, J. A. Comparison of active treatment and placebo in older Chinese patients with isolated systolic hypertension. Systolic Hypertension in China (Syst-China) Collaborative Group. *J Hypertens* **16**, 1823–1829 (1998).
Syst-Eur, Systolic Hypertension in Europe Trial	Staessen, J. A. *et al.* Randomised double-blind comparison of placebo and active treatment for older patients with isolated systolic hypertension. The Systolic Hypertension in Europe (Syst-Eur) Trial Investigators. *Lancet* **350**, 757–764 (1997).
The BBB study	Hannson, L. The BBB Study: the effect of intensified antihypertensive treatment on the level of blood pressure, side-effects, morbidity and mortality in 'well-treated' hypertensive patients. Behandla Blodtryck Bättre. *Blood Press* **3**, 248–254 (1994).
TOMHS	Neaton, J. D. *et al.* Treatment of mild hypertension study. Final results. Treatment of Mild Hypertension Study Research Group. *JAMA* **270**, 713–724 (1993).
TRANSCEND, Telmisartan Randomized Assessment Study	Telmisartan Randomised AssessmeNt Study in ACE iNtolerant subjects with cardiovascular Disease (TRANSCEND) Investigators *et al.* Effects of the angiotensin-receptor blocker telmisartan on cardiovascular events in high-risk patients intolerant to angiotensin-converting enzyme inhibitors: a randomised controlled trial. *Lancet* **372**, 1174–1183 (2008).
UKPDS UK, Prospective Diabetes Study (UKPDS 38)	UK Prospective Diabetes Study Group. Tight blood pressure control and risk of macrovascular and microvascular complications in type 2 diabetes: UKPDS 38. *BMJ* **317**, 703–713 (1998).
VALISH, Valsartan in Eldery Isolated Systolic Hypertension Study	Ogihara, T. *et al.* Target blood pressure for treatment of isolated systolic hypertension in the elderly. *Hypertension* **56**, 196–202 (2010).

Supplementary Table 4. Mean relative risk measures of IHD risk across systolic blood pressure exposure

Relative risk based on the mean relative risk function (95% UI accounting for between-study heterogeneity), presented at every 10 mmHg of systolic blood pressure from 100 to 200 mmHg. The mean RRs are calculated in comparison to a reference SBP level of 100 mmHg. IHD = ischemic heart disease. RR = relative risk. UI = uncertainty interval.

SBP level (mmHg)	RR (95% UI)
100	1 (ref.)
107.5*	1.06 (1.06 to 1.07)
110	1.12 (1.10 to 1.13)
120	1.39 (1.34 to 1.44)
130	1.81 (1.70 to 1.93)
140	2.38 (2.17 to 2.62)
150	3.11 (2.75 to 3.52)
160	3.99 (3.43 to 4.63)
165*	4.48 (3.81 to 5.26)
170	4.95 (4.17 to 5.89)
180	5.66 (4.69 to 6.82)

190	6.15 (5.05 to 7.48)
200	6.64 (5.41 to 8.15)

*15th and 85th percentile of the SBP exposure.

Supplementary Table 5. Burden of proof risk function for high systolic blood pressure exposure and ischemic heart disease.

Burden of proof risk function defined as the 5th quantile risk curve (closest to null)—inclusive of between-study heterogeneity, providing a conservative estimate of effect size and evidence strength—averaged across the data dense 15th–85th percentile range (107.5 to 165 mmHg) of systolic blood pressure (SBP) exposure. The BPRF is calculated in comparison to a reference SBP level of 100 mmHg. Risk outcome score (ROS) calculated as the average log relative risk of the BPRF over the 15th–85th percentile of the SBP exposure range. Star rating summary measure of risk and evidence strength: ROS<0 yields 1 star, 0–15% risk increase yields 2 stars, >15–50% risk increase yields 3 stars, >50–85% risk increase yields 4 stars, and >85% risk increase yields 5 stars. BPRF = burden of proof risk function, ROS = risk outcome score, SBP = Systolic blood pressure.

SBP level (mmHg)	Relative risk (95% UI)	Exposure-averaged BPRF	ROS	Star rating
100	1 (ref.)			
Averaged over 107.5–165*		2.01	0.70	5

*15th and 85th percentile of the SBP exposure.

Supplementary Table 6. GATHER checklist

Item #	Checklist item	Reported on page #
**Objectives and funding**		
**1**	Define the indicator(s), populations (including age, sex, and geographic entities), and period(s) for which estimates were made.	Main text (methods and results section); Supplemental Information Section 3
**2**	List the funding sources for the work.	Main text (acknowledgement section)
**Data Inputs**		
For all data inputs from multiple sources that are synthesized as part of the study:		
**3**	Describe how the data were identified and how the data were accessed.	Main text (step 1 in methods); Extended Data Figure 1 (PRISMA 2020 flow diagram)
**4**	Specify the inclusion and exclusion criteria. Identify all ad-hoc exclusions.	Main text (step 1 in methods); Extended Data figure 1 (PRISMA 2020 flow diagram)
**5**	Provide information on all included data sources and their main characteristics. For each data source used, report reference information or contact name/institution, population represented, data collection method, year(s) of data collection, sex and age range, diagnostic criteria or measurement method, and sample size, as relevant.	Main table 1;<br>Data sources and citations for each risk-outcome pair can be found in the reference list and in Supplementary information Table 3 and can be downloaded from the Burden of Proof visualization tool: http://vizhub.healthdata.org/burden-of-proof
**6**	Identify and describe any categories of input data that have potentially important biases (e.g., based on characteristics listed in item 5).	Main text (methods and results sections)
For data inputs that contribute to the analysis but were not synthesized as part of the study:		
**7**	Describe and give sources for any other data inputs.	Not applicable
For all data inputs:		
**8**	Provide all data inputs in a file format from which data can be efficiently extracted (e.g., a spreadsheet rather than a PDF), including all relevant meta-data listed in item 5. For any data inputs that cannot be shared because of ethical or legal reasons, such as third-party ownership, provide a contact name or the name of the institution that retains the right to the data.	See Data Availability statement.<br>Data sources and citations can be downloaded from the Burden of Proof visualization tool: http://vizhub.healthdata.org/burden-of-proof
**Data analysis**		
**9**	Provide a conceptual overview of the data analysis method. A diagram may be helpful.	Main text (methods overview)
**10**	Provide a detailed description of all steps of the analysis, including mathematical formulae. This description should cover, as relevant, data cleaning, data pre-	Main text (methods section)

	processing, data adjustments and weighting of data sources, and mathematical or statistical model(s).	
**11**	Describe how candidate models were evaluated and how the final model(s) were selected.	Main text ("model validation" in methods)
**12**	Provide the results of an evaluation of model performance, if done, as well as the results of any relevant sensitivity analysis.	Main text ("sensitivity analysis" in methods); Supplementary Information Section 3. Extended Data Figures 3-9
**13**	Describe methods for calculating uncertainty of the estimates. State which sources of uncertainty were, and were not, accounted for in the uncertainty analysis.	Main text (methods section)
**14**	State how analytic or statistical source code used to generate estimates can be accessed.	Code is accessible on GitHub: https://github.com/ihmeuw-msca/burden-of-proof
**Results and Discussion**		
**15**	Provide published estimates in a file format from which data can be efficiently extracted.	Risk-outcome scores; star ratings; risk curves with all data points, trimmed data points, and conventional and conservative uncertainty intervals; and an interpretation of the findings are available for all risk-outcome pairs at http://vizhub.healthdata.org/burden-of-proof
**16**	Report a quantitative measure of the uncertainty of the estimates (e.g. uncertainty intervals).	UIs given in all main text figures; online viz tool: http://vizhub.healthdata.org/burden-of-proof
**17**	Interpret results in light of existing evidence. If updating a previous set of estimates, describe the reasons for changes in estimates.	Main text (discussion)
**18**	Discuss limitations of the estimates. Include a discussion of any modelling assumptions or data limitations that affect interpretation of the estimates.	Main text (limitations section in discussion)

Supplementary Table 7. Causal criteria extraction template

Category	Variable	Definition
Source	seq	
	underlying_nid	Underlying NID: Enter the underlying NID of the study (if applicable). Always talk to a data indexer if you don't know if an underlying NID is needed. They may be used for meta-analyses, certain database sources, and in some other specific cases.
	nid	Found in GHDx, created through the epi form, or created by Data Indexer
	field_citation_value	IHME Zotero format or if source has NID, citation info from GHDx
	file_path	optional; full file path of article; Only needed if source doesn't have NID, to facilitate NID creation.
R-O pair	risk	Risk: Select the risk factor, if not listed here, contact the causal criteria team
	risk_mapping	The relationship between study definition of risk and GBD definition of risk for a particular effect size
	outcome	Outcome: Select the outcome.
	outcome_mapping	The relationship between study definition of outcome and GBD definition of outcome for a particular effect size
Location	location_name	Location name (from locations tab). Do a fast double-click in this field to get the drop-down menu, then start typing the location_name. For location_names with special characters, you may need to use the scroll bar.
	location_id	Autopopulated from location_name
	rep_geography	Were the study participants representative of the geography? 1=yes, 0=no
	rep_selection_criteria	If rep_geography is 0, please specify the selection criteria of the study that is used in the analysis
	rep_prevalent_disease	Is the study aiming to evaluate the risk or mortality of people who have already developed the outcome? 1=yes 0=no (i.e. yes if for SBP-IHD paper, all participants have IHD at baseline and the paper is looking at mortality due to SBP, no if for SBP-IHD paper the participants have other prevalent diseases)
Study Population	year_start_study	Year the study was started. If not specified, leave blank
	year_end_study	Year the study was finished (including most recent follow up). If not specified, leave blank
	age_start	Ages from 1 and above must be entered as an integer. Ages <1 can be entered as decimal values, e.g., 3 days = 3/365.
	age_end	Ages from 1 and above must be entered as an integer. Ages <1 can be entered as decimal values, e.g., 3 days = 3/365.

Category	Variable	Definition
	age_mean	Mean age
	age_sd	SD of age
	age_issue	0 = no issue flagged; 1 = issue flagged for modeler; always include explanatory notes the note_SR column
	percent_male	What percent of the population is male (0-1), if pop is all female then it would be 0
	sex_issue	sex_issue
Study Design	design	Study design: Specify the design of the study
	study_name	Study Name: Enter the name of the study (e.g., Nurses' Health Study), if provided. Do not enter the title of the article.
Exposure	exp_assess_level	Level of exposure assessment: The exposure was assessed…
	exp_instrument	Exposure assessment instrument: Specify the name of the exposure assessment instrument. For self-reported exposures, please specify the name of the questionnaire e.g., International Physical Activity Questionnaire (IPAQ). If more than one instrument specify all
	exp_assess_period	What was the frequency of exposure assessment?
	exp_assess_num	If multiple, specify the number of times that exposure was assessed (excluding baseline)
	exp_method_1	Please specify the method of exposure assessment. If there are more than 1, please add in the next columns labeled "exp_method_2".
	exp_method_2	Please specify the method of exposure assessment. If there are more than 2, please add in the next columns labeled "exp_method_3".
	exp_method_3	Please specify the method of exposure assessment.
	exp_recall_period	This field describes the unit of exposure recall used in data collection ONLY for self-report. Select the correct option from the drop-down menu. If the unit is days, weeks, months, or years, please enter the number in exp_recall_period_value (next column). If the unit is 'lifetime', nothing needs to be entered in exp_recall_period_value. For example, if the study said the recall period was 4 weeks, enter 4 in exp_recall_period_value, and 'weeks' in the field exp_recall_period. If 'other' is selected, please describe in exp_recall_period_other
	exp_recall_period_value	If you entered days, weeks, months, or years in the field 'exp_recall_period', please enter the corresponding integer in this field. For example, if the study said the recall period was 4 weeks, enter 4 in exp_recall_period_value, and 'weeks' in the field exp_recall_period.
	exp_recall_period_other	If 'other' was selected in exp_recall_period, please describe the exposure recall period that the study specified (e.g., recall of exposure from 12 to 18 years).
	exp_type	Which form of the exposure was included in relative risk estimation analysis?
Outcome	outcome_def	Outcome definition: Provide a brief description of the outcome as reported in the study.
	outcome_type	Outcome type: please specify if the outcome definition included incidence of or mortality from a disease endpoint
	outcome_assess_1	Method of outcome assessment: Specify the method of assessment of the study outcome. If more than 1 are appropriate, enter additional methods in the next column labeled "outcome_assess_2"
	outcome_assess_2	Method of outcome assessment: Specify the method of assessment of the study outcome. If more than 2 are appropriate, enter additional methods in the next column labeled "outcome_assess_3"
	outcome_assess_3	Method of outcome assessment: Specify the method of assessment of the study outcome.
Follow up	duration_fup_measure	Type of follow up measure (i.e. mean, median, max, min)
	duration_fup_units	Units of follow up duration
	value_of_duration_fup	Enter the length of participant follow-up.
Confounders	confounders_age	If controlled for in the relative risk estimation analysis, mark 1 for yes. Mark 0 for no
	confounders_sex	If controlled for in the relative risk estimation analysis, mark 1 for yes. Mark 0 for no
	confounders_education	If controlled for in the relative risk estimation analysis, mark 1 for yes. Mark 0 for no
	confounders_income	If controlled for in the relative risk estimation analysis, mark 1 for yes. Mark 0 for no
	confounders_smoking	If controlled for in the relative risk estimation analysis, mark 1 for yes. Mark 0 for no

Category	Variable	Definition
	confounders_alcohol_use	If controlled for in the relative risk estimation analysis, mark 1 for yes. Mark 0 for no
	confounders_physical_activity	If controlled for in the relative risk estimation analysis, mark 1 for yes. Mark 0 for no
	confounders_dietary_components	If controlled for in the relative risk estimation analysis, mark 1 for yes. Mark 0 for no
	confounders_bmi	If controlled for in the relative risk estimation analysis, mark 1 for yes. Mark 0 for no
	confounders_hypertension	If controlled for in the relative risk estimation analysis, mark 1 for yes. Mark 0 for no
	confounders_diabetes	If controlled for in the relative risk estimation analysis, mark 1 for yes. Mark 0 for no
	confounders_hypercholesterolemia	If controlled for in the relative risk estimation analysis, mark 1 for yes. Mark 0 for no
	confounders_other	For other confounders that are not listed, list here
Effect Size	page_num_effect_size	Page number (where you found effect_size) from literature, or survey question where you found effect size; Use page number(s) of article, not page # of pdf
	effect_size_measure	Effect size measure: Specify the measure of effect size
	effect_size	Effect size estimate: Provide the effect size estimate
	lower	Provide the lower limit of the confidence interval. Enter on a "per 1" basis. (If the CI is reported as a percent, you must convert to a decimal.) These 3 fields must all be filled in if any of them are filled in: lower, upper, uncertainty_type_value.
	upper	Provide the upper limit of the confidence interval. Enter on a "per 1" basis. (If the CI is reported as a percent, you must convert to a decimal.) These 3 fields must all be filled in if any of them are filled in: lower, upper, uncertainty_type_value.
	CI_uncertainty_type_value	This field is required if 'lower' & 'upper' are entered. This column represents the confidence level which is reported at (Eg. 95, 90, 99). These 3 fields must all be filled in if any of them are filled in: lower, upper, uncertainty_type_value.
	nonCI_uncertainty_value	Numerical value of the nonCI_uncertainty_type entered in that column. For example, if SD=5.3, you'd put 5.3 in this column, and choose SD from the drop-down menu in nonCI_uncertainty_type.
	nonCI_uncertainty_type	Enter SE or SD if appropriate. For example, if SD=5.3, you'd put 5.3 in nonCI_uncertainty_value, and choose SD from the drop-down menu in this column (nonCI_uncertainty_type).
	uncertainty_issue	Mark with a 1 if no uncertainty is reported, if some sort of uncertainty is reported, mark 0
	subgroup_analysis	1 if RR is from main analysis (all participants), 0 if sub-analysis (only males, or among a specific age group, etc.)
	subgroup_analysis_free_text	If a sub-analysis, describe it (i.e., age, sex, etc.)
	effect_size_multi_location	1 if the reported effect size is from a multi-country study and only one effect size has been reported for all locations, otherwise 0
	effect_size_multi_location_specify	Which geography level is the RR for
	pooled_cohort	1 if the reported effect size is from a pooled analysis and only pooled effect size has been reported, otherwise 0
	dose_response	Does the study support a dose-response relationship between the exposure and the outcome? (1= yes, 0=no)
	dose_response_detail	If "1" was specified in the dose_response field, please specify in this field the type of evidence supporting the dose-response relationship. For example, "statistically significant p value for linear trend".
Cohorts	cohort_person_years_exp	Please specify the person-years of follow up in the exposed group
	cohort_person_years_unexp	Please specify the person-years of follow up in the unexposed group
	cohort_person_years_total	Enter the total person-years of follow-up if person-years of follow up in exposed and unexposed not reported
	cohort_number_events_exp	Please specify the number of events in the exposed group
	cohort_number_events_unexp	Please specify the number of events in the unexposed group
	cohort_number_events_total	Enter the total number of events/cases if number of events in exposed and unexposed not reported
	cohort_sample_size_exp	Please specify the number of people in the exposed group if person-years of follow up in exposed not reported

Category	Variable	Definition
	cohort_sample_size_unexp	Please specify the number of people in the unexposed group if person-years of follow up in unexposed not reported
	cohort_sample_size_total	Please specify the number of people included in the analysis if total person-years of follow up is not reported
	cohort_dropout_rate	Dropout rate: Specify the dropout rate (%) at the end of the study. Enter on a "per 1" basis. For example: 23% is entered as .23.
	cohort_dropout_assess	Specify how dropout rate was defined in the study.
	cohort_exposed_def	Exposed group definition: Provide a brief description of the exposed group (i.e., the comparison group) as used in estimation of the relative risk (e.g., never smokers)
	cohort_exp_unit_rr	Exposure unit (for continuous risks): Specify the unit of exposure (e.g., grams/day).
	cohort_exp_level_rr	Exposure level in the exposed group (for continuous risks): Specify the mean/median level of exposure in the exposed group.
	cohort_unexp_def	unexposed group definition: Provide a brief description of the unexposed group (i.e., the comparison group) as used in estimation of the relative risk (e.g., never smokers)
	cohort_unexp_unit_rr	Exposure unit (for continuous risks): Specify the unit of exposure (e.g., grams/day) for the unexposed group
	cohort_unexp_level_rr	Exposure level in the unexposed group (for continuous risks): Specify the mean/median level of exposure in the unexposed group.
	cohort_exp_level_dr	Exposure level in for dose-repose RRs (for continuous risks): If the study reports dose-repose RR, please specify the level of exposure for the reported RR
Case-control	cc_community	Were the controls selected from the community? 1 = yes, 0=no
	cc_cases	Number of cases
	cc_control	Number of controls
	cc_exposed_def	Exposed group definition: Provide a brief description of the exposed group for which the relative risk is reported (e.g., current smokers)
	cc_exp_unit_rr	Exposure unit (for continuous risks): Specify the unit of exposure (e.g., grams/day).
	cc_exp_level_rr	Exposure level in the exposed group (for continuous risks): Specify the mean/median level of exposure in the exposed group.
	cc_unexposed_def	Unexposed group definition: Provide a brief description of the unexposed group (i.e., the comparison group) as used in estimation of the relative risk (e.g., never smokers)
	cc_unexp_unit_rr	Unexposed unit (for continuous risks)
	cc_unexp_level_rr	Exposure level in the unexposed group (for continuous risks): Specify the mean/median level of exposure in the unexposed group.
	cc_exp_level_dr	Exposure level in for dose-repose RRs (for continuous risks): If the study reports dose-repose RR, please specify the level of exposure for the reported RR
Trials	int_intervention_description	Intervention definition: Provide a brief description of the intervention as reported in the study.
	int_control_description	Control definition: Provide a brief description of the control as reported in the study.
	int_intervention_multi_rf	Does this intervention simultaneously target more than one risk? (1=yes, 0=no)
	int_intervention_multi_rf_specify	Specify the risks that are targeted by the intervention
	int_intervention_level	Level of intervention: The intervention was implemented …
	int_adhere_assess	Specify how adherence was defined in the study.
	int_adhere_rate_intervention	Adherence rate in the intervention group; Enter on a "per 1" basis. For example: 23% is entered as .23.
	int_adhere_rate_control	Adherence rate in the control group; Enter on a "per 1" basis. For example: 23% is entered as .23.
	int_dropout_rate_intervention	Dropout rate in the intervention group: Specify the dropout rate (%) at the end of the study. Enter on a "per 1" basis. For example: 23% is entered as .23.
	int_dropout_rate_control	Dropout rate in the control group: Specify the dropout rate (%) at the end of the study. Enter on a "per 1" basis. For example: 23% is entered as .23.
	int_dropout_assess	Specify how dropout rate was defined in the study.
	int_blinding	For interventional studies. Blinding: The trial was … (select 1)
	int_exp_unit	For trials, specify the unit of exposure (e.g., mmol/l)

Category	Variable	Definition
	int_baseline_exp_int	For trials, specify the exposure level in the intervention group at baseline
	int_baseline_exp_comp	For trials, specify the exposure level in the comparison group at baseline
	int_fup_exp_int	For trials, specify the exposure level in the intervention group at the end of the follow-up time
	int_fup_exp_comp	For trials, specify the exposure level in the comparison group at the end of follow up time
	int_fup_exp_int_difference	For trials, please specify the difference of exposure level between baseline and follow up time for the intervention group
	int_fup_exp_comp_difference	For trials, please specify the difference of exposure level between baseline and follow up time for the comparison group
	int_person_years_int	Please specify the number of person years of follow up for the intervention group
	int_person_years_comp	Please specify the number of person years of follow up in the comparison group
	int_number_events_int	For trials, specify the number of cases in the intervention group at the end of follow up
	int_number_events_comp	For trials, specify the number of cases in the control group at the end of follow up
	int_sample_size_int_group_baseline	For trials, specify the sample size in the intervention group at baseline
	int_sample_size_comparison_group_baseline	For trials, specify the sample size in the comparison group at baseline
	int_sample_size_int_group_follow_up	For trials, specify the sample size in the intervention group at the end of the follow-up time
	int_sample_size_comparison_group_follow_up	For trials, specify the sample size in the comparison group at the end of follow up time
Other	note_modeler	For modelers only, audience is modeler, not for correspondence
	note_sr	notes related to extraction, including assumptions, data adjustment, problems with source, any other notes that may be relevant, etc.
	extractor	Identifier (uwnet id) of person who extracted the data
Custom	custom_exp_meas_num	If the exposure level was assessed multiple times at a given time point (e.g., systolic blood pressure), specify the number of measurements at each time point.
	custom_exp_biomarker	If the exposure level was assessed via a biomarker, specify the full name of the biomarker.
	custom_exp_kilometer	Specify the geographical unit of measurement in kilometer (if applicable, e.g., satellite data).
	custom_exp_level_lower	If don't have a mean/midpoint exposure level can use this column in conjecture with the custom_exp_level_upper to enter in a range
	custom_exp_level_upper	If don't have a mean/midpoint exposure level can use this column in conjecture with the custom_exp_level_lower to enter in a range
	custom_unexp_level_lower	If don't have a mean/midpoint exposure level can use this column in conjecture with the custom_outcome_level_upper to enter in a range
	custom_unexp_level_upper	If don't have a mean/midpoint exposure level can use this column in conjecture with the custom_outcome_level_lower to enter in a range
	custom_prospective_lag	Specify lag time between exposure assessment and outcome
	custom_age_demographer	A binary flag to identify if ages are presented in demographer notation or not in the source. This value is currently not used to adjust any age_start or age_end values, but in the future, that is the intention; 0 = article does not use demographer notation (4 = 4.00 not 4.99); 1 = article uses demographer notation (4=4.99 not 4.00)
	custom_bmi_menopause_free_text	Free text field for bmi team
	custom_cvd_outcome	Used for mapping cvd outcomes, free text field
	custom_dm_type	Used for documenting diabetes type
	custom_dm_case_defn	Used for documenting diabetes definitions, free text
	custom_pmid	Document PubMed id
	custom_cvd_rep_high_risk	CVD-specific, binary, if the study only includes people at high risk for CVD (1 for example if it is only among diabetics)
	custom_drug_class	Class of drug being used in intervention, free text
	custom_outcome_primary	Outcome is the primary outcome of RCT (1=yes, 0=no)
	custom_outcome_prespecified	Outcome is the prespecified outcome of RCT (1=yes, 0=no)
	custom_multipollutant	Are any other pollutants controlled for in the model? 0=no, 1=yes
	custom_pollutants_controlled	If custom_multipollutant=1, list the pollutants controlled for

Category	Variable	Definition
	custom_PM2.5_model_type	Describe the model used for exposure
	custom_assign_method	How do researchers assign participants to exp? (ex: by home address, by city, nearest zipcode centroid, etc.)
	custom_PM2.5_def	What metric are they using to measure PM2.5 (ex: mean of annual PM2.5 averages for 35-1 year prior to study)
	custom_lag	Do the authors take into account lag? If so, how?
	custom_PM2.5_min	All of these have to do with the spread of the PM2.5 exposure covered by the study. Minimum
	custom_PM2.5_5th	5th percentile
	custom_PM2.5_25th	25th percentile
	custom_PM2.5_50th	Median/50thpercentile
	custom_PM2.5_75th	75th percentile
	custom_PM2.5_95th	95th percentile
	custom_PM2.5_max	Maximum
	custom_PM2.5_mean	Mean
	custom_PM2.5_stddev	Standard deviation
	custom_PM2.5_other_measure	Any other measures of the distribution of PM2.5 amongst participants?
	custom_PM2.5_other_measure_description	If so, what are they? (e.g., 10th, 90th, IQR)

Supplementary Table 8. Study covariates assessed in the analysis

Domain	Covariate name	Definition	Completeness (%)*
Representativeness	Representativeness	Scored 0 for studies whose results are likely generalizable to the general population because the sample was based on the general population with reasonable exclusions for pre-existing disease states and 1 for analyses in sub-groups such as high-risk groups	100
Exposure measurement	Exposure population	Scores 0 for individual level exposure and 1 for population level exposure	100
	Exposure self-report	Scores 0 for measurements based on assays, tests or physician observation and 1 for self-report	100
	Exposure study	Scores 0 if exposure was measured multiple times and 1 for only a baseline measurement. In RCTs score the study as 0	100
Outcome measurement	Outcome self-report	Scores 0 if the outcome measurement was based on death certificates or medical records and scores 1 if based on self-report	100
	Outcome unblinded	Scores 0 if the assessment of outcome is blind to the individual level of exposure or vice versa for outcome and 1 if unblinded	100 (of RCTs)
	Outcome definition	One dummy variable per definition of the outcome including ischemic heart disease, coronary heart disease angina, revascularization, was generated.	100
	Outcome type	Incidence or mortality	100
Reverse causation	Reverse causation	Scores 0 if there is minimal or no risk of reverse causation and 1 if there is a risk of reverse causation.	64
Control for confounding	Confounding_nonrandom	Scores 0 for a randomized study and 1 for a non-randomized study	95.3
	Confoundng uncontrolled	Scores 0 for randomization or for a non-randomized study but the outcome is controlled for all major known confounders including age, sex, smoking, education, income, body mass index and/or cholesterol measurements and other critical determinants of that outcome. Scores 1 for non-randomized with control for age, sex, and other critical determinants of that outcome. Scores 2 if only controls for age and sex and select determinants	44.4
	Blinding	Scores 0 for RCTs double or triple blinded, 1 for single blinding and 2 for no blinding	100
Selection bias	Selection bias	Scores 0 for greater than 95% follow-up, scores 1 for follow up of 85-95% and scores 2 for less than 85% follow up	92
Study type	Trials	Scores 0 for RCTs and 1 for other study types	100
	Cohorts	Scores 0 for cohorts or pull cohorts and 1 for other study types	100
Risk measurement	Risk measurement	Dummy variables were created to identify studies reporting hazard ratios, relative risks and odds ratio.	100

*Completeness of a covariate was defined as the percentage of studies included in the analysis reporting relevant information.

Section 2: Data source identification

The data used for this study includes randomized control trials (RCTs) and pooled cohort studies. More detailed information on data inputs is provided in the online viz tool: http://vizhub.healthdata.org/burden-of-proof.

Section 2.1 Literature studies

We conducted a literature review to obtain input data from randomized control trials evaluating the relationship between systolic blood pressure levels and ischemic heart disease. We also searched citation lists of the most recent systematic reviews of clinical trials.

Section 2.1.1 PubMed search

A literature search was performed on PubMed using the following search string. Inclusion and exclusion criteria are described in the Methods section of the main text.

Search string

("blood pressure"[Title/Abstract] OR "blood pressure"[MeSH Terms] OR "anti-hypertensive"[Title/Abstract] OR "blood pressure-lowering"[Title/Abstract] OR "blood pressure-lowering"[Title/Abstract] OR "antihypertensive agents"[MeSH Terms] OR "Ambrisentan"[Text Word] OR "Bosentan"[Text Word] OR "Diazoxide"[Text Word] OR "iloprost"[Text Word] OR "Minoxidil"[Text Word] OR "Sildenafil"[Text Word] OR "sodium nitroprusside"[Text Word] OR "Tadalafil"[Text Word] OR "Methyldopa"[Text Word] OR "Clonidine"[Text Word] OR "moxonidine"[Text Word] OR "Guanethidine"[Text Word] OR "Doxazosin"[Text Word] OR "Indoramin"[Text Word] OR "Prazosin"[Text Word] OR "Terazosin"[Text Word] OR "Phenoxybenzamine"[Text Word] OR "Phentolamine"[Text Word] OR "Atenolol"[Text Word] OR "Metoprolol"[Text Word] OR "Pindolol"[Text Word] OR "Timolol"[Text Word] OR "Oxprenolol"[Text Word] OR "Nebivolol"[Text Word] OR "Nadolol"[Text Word] OR "Labetalol"[Text Word] OR "Celiprolol"[Text Word] OR "Carvedilol"[Text Word] OR "Bisoprolol"[Text Word] OR "Bisoprolol"[Text Word] OR "Propranolol"[Text Word] OR "Hydrochlorothiazide"[Text Word] OR "Trichlormethiazide"[Text Word] OR "Spironolactone"[Text Word] OR "Chlortalidone"[Text Word] OR "Indapamide"[Text Word] OR "Captopril"[Text Word] OR "Cilazapril"[Text Word] OR "Enalapril"[Text Word] OR "Enalapril"[Text Word] OR "Imidapril"[Text Word] OR "Lisinopril"[Text Word] OR "Moexipril"[Text Word] OR "Perindopril"[Text Word] OR "Quinapril"[Text Word] OR "Ramipril"[Text Word] OR "Trandolapril"[Text Word] OR "Azilsartan"[Text Word] OR "Candesartan"[Text Word] OR "Eprosartan"[Text Word] OR "Irbesartan"[Text Word] OR "Losartan"[Text Word] OR "Olmesartan"[Text Word] OR "Telmisartan"[Text Word] OR "Valsartan"[Text Word] OR "Aliskiren"[Text Word] OR "Amlodipine"[Text Word] OR "Diltiazem"[Text Word] OR "Felodipine"[Text Word] OR "Isradipine"[Text Word] OR "Lacidipine"[Text Word] OR "Lercanidipine"[Text Word] OR "Nicardipine"[Text Word] OR "Nifedipine"[Text Word] OR "Nisoldipine"[Text Word] OR "Verapamil"[Text Word] OR "Nitrendipine"[Text Word]) AND "clinical trial"[Publication Type] AND 2018/2/1:2020/4/1[Date - Publication]

Section 3: Sensitivity analysis

Section 3.1. Model results based on input data: testing cohort studies vs RCTs

To estimate the shape of the risk-outcome relationship directly from the data and to validate using evidence from both prospective cohort studies and RCTs, we performed a sensitivity analysis as follows. We first modeled a non-linear curve including only data from cohort studies without monotonicity constraints, not assuming a log-linear relationship. We then fit a similar model from RCT data only (see figures below). We decided to use all available data combining evidence from different study designs given that 1) RCTs are a rich source of exposure and outcome information, 2) most of the SBP population evidence typically comes from populations at high IHD risk and/or with treated high blood pressure, 3) cohort studies account for populations with normal and below normal SBP levels, and 4) the shape of the relationship without constraints was remarkably similar across all models. See Extended Data Figures 6–9 for results.
