# Supplementary material for: Effects of elevated systolic blood pressure on ischemic heart disease: a Burden of Proof study
Source: Nat Med. 2022 Oct 10;28(10):2056–65. doi: 10.1038/s41591-022-01974-1 (PMC9556328; doi:10.1038/s41591-022-01974-1)
Supplement: Supplementary file 2 — Reporting Summary [file 41591_2022_1974_MOESM2_ESM.pdf]

nature portfolio

Corresponding author(s): Christian Razo

Last updated by author(s): Jul 22, 2022

Reporting Summary

Nature Portfolio wishes to improve the reproducibility of the work that we publish. This form provides structure for consistency and transparency in reporting. For further information on Nature Portfolio policies, see our Editorial Policies and the Editorial Policy Checklist.

Statistics

For all statistical analyses, confirm that the following items are present in the figure legend, table legend, main text, or Methods section.

n/a	Confirmed	
☐	☒	The exact sample size (*n*) for each experimental group/condition, given as a discrete number and unit of measurement
☐	☒	A statement on whether measurements were taken from distinct samples or whether the same sample was measured repeatedly
☐	☒	The statistical test(s) used AND whether they are one- or two-sided<br>*Only common tests should be described solely by name; describe more complex techniques in the Methods section.*
☐	☒	A description of all covariates tested
☐	☒	A description of any assumptions or corrections, such as tests of normality and adjustment for multiple comparisons
☐	☒	A full description of the statistical parameters including central tendency (e.g. means) or other basic estimates (e.g. regression coefficient) AND variation (e.g. standard deviation) or associated estimates of uncertainty (e.g. confidence intervals)
☐	☒	For null hypothesis testing, the test statistic (e.g. *F*, *t*, *r*) with confidence intervals, effect sizes, degrees of freedom and *P* value noted<br>*Give P values as exact values whenever suitable.*
☐	☒	For Bayesian analysis, information on the choice of priors and Markov chain Monte Carlo settings
☒	☐	For hierarchical and complex designs, identification of the appropriate level for tests and full reporting of outcomes
☐	☒	Estimates of effect sizes (e.g. Cohen's *d*, Pearson's *r*), indicating how they were calculated

Our web collection on statistics for biologists contains articles on many of the points above.

Software and code

Policy information about availability of computer code

Data collection	No primary data collection was carried out for this analysis.
Data analysis	All code used for these analyses is publicly available online (https://github.com/ihmeuw-msca/burden-of-proof). This includes code for the meta-regression engine, the model specification interface, both parts of the data processing, and risk-specific custom code, as appropriate. Analyses were carried out using R version 3.6.1, Python version 3.8, and Stata version 17.<br><br>To validate key aspects of the meta-regression model used in this analysis, the following packages were used, as described in Zheng et al: metafor (R package available for download at https://www.jstatsoft.org/article/view/v036i03) and dosmesreta (R package available for download at https://www.jstatsoft.org/article/view/v072c01).

For manuscripts utilizing custom algorithms or software that are central to the research but not yet described in published literature, software must be made available to editors and reviewers. We strongly encourage code deposition in a community repository (e.g. GitHub). See the Nature Portfolio guidelines for submitting code & software for further information.

Data

Policy information about availability of data

All manuscripts must include a data availability statement. This statement should provide the following information, where applicable:

- Accession codes, unique identifiers, or web links for publicly available datasets
- A description of any restrictions on data availability
- For clinical datasets or third party data, please ensure that the statement adheres to our policy

The findings from this study were produced using data available in the published literature. Data sources and citations for each risk-outcome pair can be downloaded using the "download" button on each risk curve page currently available at https://vizhub.healthdata.org/burden-of-proof/. Study characteristics and citations for all input data used in the analyses are also provided in Table 2 and Supplementary Information Table S5.

Human research participants

Policy information about studies involving human research participants and Sex and Gender in Research.

Reporting on sex and gender	No primary data collection was carried out for this analysis, so the study does not involve human research participants. As stated in the methods overview, our estimates are not specific to or disaggregated by specific populations, including by sex: neither sex nor gender were considered in the study design. We included all available data regardless of how or if the input study collected and reported data by sex or gender. Although most of the studies we extracted data from included information about the self-reported sex of the participants, they rarely reported relative risk estimates by sex, which precluded us from performing any sex- or gender-based analyses.
Population characteristics	Primary data collection was not carried out for this meta-analysis. Covariate-relevant population characteristics of the source studies included are: past and current CVD-related diagnoses, age, and intervention applied. See Table 2.
Recruitment	No primary data collection was carried out for this analysis, so we did not recruit participants.
Ethics oversight	The Global Burden of Diseases, Injuries, and Risk Factors Study—which this study falls under—used de-identified data, and the waiver of informed consent was reviewed and approved by the University of Washington Institutional Review Board (study number 9060) through November 1, 2022.

Note that full information on the approval of the study protocol must also be provided in the manuscript.

Field-specific reporting

Please select the one below that is the best fit for your research. If you are not sure, read the appropriate sections before making your selection.

☒ Life sciences ☐ Behavioural & social sciences ☐ Ecological, evolutionary & environmental sciences

For a reference copy of the document with all sections, see nature.com/documents/nr-reporting-summary-flat.pdf

Life sciences study design

All studies must disclose on these points even when the disclosure is negative.

Sample size	No sample size calculation was performed for this meta-analysis; all available datasets meeting inclusion criteria or included. As reported in the results section, 1,492,452 unique participants and 12,210 IHD events were included in this meta-analysis.
Data exclusions	RCTs were eligible for inclusion if: 1) participants were randomly allocated to treatment vs control, or treatment target groups; 2) relative risk (RR) estimates (risk ratios, incidence rate ratios, odds ratios or hazard ratio) for incidence or mortality of an outcome of interest were reported for each group; 3) mean pre-and post-intervention (or, alternatively, baseline and follow-up for cohort studies) SBP levels were reported for each group; and 4) outcomes of interest included myocardial infarction, angina, coronary heart disease, heart failure, major adverse cardiovascular events, or revascularization cases.<br><br>Studies were excluded if: 1) they were duplicates; 2) they did not report SBP levels; 3) they did not report outcomes relevant to this analysis, e.g., focused primarily on secondary hypertension or sudden cardiac death, severe arrhythmia, all-cardiovascular mortality, or all-cause mortality; and 4) they were head-to-head comparisons of different drug classes or trials of alternate blood pressure-lowering pharmacotherapies that were not intended to achieve identified target SBP levels.
Replication	This is a meta-analysis of existing studies with many years of cohort and other data. When re-applying the method to the same data, we get the same results.
Randomization	This analysis is a meta-analysis of existing studies and thus, there were no experimental groups.
Blinding	N/A. Blinding was not relevant to this study, as we did not collect primary data.

Reporting for specific materials, systems and methods

We require information from authors about some types of materials, experimental systems and methods used in many studies. Here, indicate whether each material, system or method listed is relevant to your study. If you are not sure if a list item applies to your research, read the appropriate section before selecting a response.

Materials & experimental systems

n/a	Involved in the study
☒	☐ Antibodies
☒	☐ Eukaryotic cell lines
☒	☐ Palaeontology and archaeology
☒	☐ Animals and other organisms
☒	☐ Clinical data
☒	☐ Dual use research of concern

Methods

n/a	Involved in the study
☒	☐ ChIP-seq
☒	☐ Flow cytometry
☒	☐ MRI-based neuroimaging
